# Predictive Models of Genetic Redundancy in *Arabidopsis thaliana*

**DOI:** 10.1093/molbev/msab111

**Published:** 2021-04-19

**Authors:** Siobhan A Cusack, Peipei Wang, Serena G Lotreck, Bethany M Moore, Fanrui Meng, Jeffrey K Conner, Patrick J Krysan, Melissa D Lehti-Shiu, Shin-Han Shiu

**Affiliations:** 1 Cell and Molecular Biology Program, Michigan State University, East Lansing, MI, USA; 2 Department of Plant Biology, Michigan State University, East Lansing, MI, USA; 3 Department of Computational Mathematics, Science and Engineering, Michigan State University, East Lansing, MI, USA; 4 Department of Botany, University of Wisconsin-Madison, Madison, WI, USA; 5 Ecology, Evolution, and Behavior Program, Michigan State University, East Lansing, MI, USA; 6 Kellogg Biological Station, Michigan State University, East Lansing, MI, USA; 7 Department of Horticulture, University of Wisconsin-Madison, Madison, WI, USA

**Keywords:** genetic redundancy, molecular evolution, machine learning

## Abstract

Genetic redundancy refers to a situation where an individual with a loss-of-function mutation in one gene (single mutant) does not show an apparent phenotype until one or more paralogs are also knocked out (double/higher-order mutant). Previous studies have identified some characteristics common among redundant gene pairs, but a predictive model of genetic redundancy incorporating a wide variety of features derived from accumulating omics and mutant phenotype data is yet to be established. In addition, the relative importance of these features for genetic redundancy remains largely unclear. Here, we establish machine learning models for predicting whether a gene pair is likely redundant or not in the model plant *Arabidopsis thaliana* based on six feature categories: functional annotations, evolutionary conservation including duplication patterns and mechanisms, epigenetic marks, protein properties including posttranslational modifications, gene expression, and gene network properties. The definition of redundancy, data transformations, feature subsets, and machine learning algorithms used significantly affected model performance based on holdout, testing phenotype data. Among the most important features in predicting gene pairs as redundant were having a paralog(s) from recent duplication events, annotation as a transcription factor, downregulation during stress conditions, and having similar expression patterns under stress conditions. We also explored the potential reasons underlying mispredictions and limitations of our studies. This genetic redundancy model sheds light on characteristics that may contribute to long-term maintenance of paralogs, and will ultimately allow for more targeted generation of functionally informative double mutants, advancing functional genomic studies.

## Introduction

Genetic redundancy, which refers to multiple genes that perform the same function, has been defined in many ways since the mid-1900s (Gabriel 1960). An early study of genetic redundancy in *Saccharomyces cerevisiae* discussed it in the context of unlinked genes encoding enzymes catalyzing the same reaction (Mortimer 1969). A later study took a broader view of genetic redundancy, with the degree of redundancy ranging from “complete redundancy” among genes with housekeeping functions to “partial overlap of function” among genes with primarily regulatory functions (Pickett and Meeks-Wagner 1995). In studies from a number of model organisms, multiple examples of what is considered genetic redundancy have been given, including: genes derived from convergent evolution encoding enzymes that perform the same function (Pickett and Meeks-Wagner 1995); biochemical pathways that are redundant due to interconnected metabolic networks (Weintraub 1993); and genes from the same family (paralogs) that maintain some of the same functionality (Kempin et al. 1995). Discussions of genetic redundancy in recent literature mostly encompass this last definition, where a duplication event results in multiple copies of a gene that retain overlapping functions (e.g., Chen et al. 2010, Bolle et al. 2013, Rutter et al. 2017). Practically, genetic redundancy is commonly observed as a single gene knockout mutant that shows no phenotype or a mild phenotype compared with a wild-type organism, with a double or higher-order mutant showing a more severe phenotype.

After a gene is duplicated, selection may be relaxed on each copy, allowing accumulation of mutations, which can lead to pseudogenization of one of the duplicates (Brookfield 1992); thus, the presence of genetically redundant paralogs long after the duplication event would seem to be an evolutionary paradox (Nowak et al. 1997). In spite of this, the literature is replete with examples of genetic redundancy, and many redundant genes in species such as *S. cerevisiae* and *Caenorhabditis elegans* originated from duplication events that happened over 600 million years ago (Ma; Vavouri et al. 2008). At least two mechanisms may explain how this is possible. Redundant copies can be retained for a long time due to the slow pace of genetic drift in large populations. Based on a few key assumptions, it is estimated that a mutation deleterious to the function of a duplicate copy could take 0.75–5 Ma to be fixed in *Arabidopsis thaliana* (Panchy et al. 2016). However, this cannot account for the apparent redundancy among paralogs from the most recent whole-genome duplication (WGD) that occurred in the Arabidopsis lineage approximately 50 Ma (Bowers et al. 2003). Another possibility is that genetic redundancy is selected for due to its ability to buffer the effect of a deleterious mutation in one paralog (Zhang 2012). The issue is that such a mechanism requires selection based on future needs, which is counter to our understanding of evolution. A mathematical model has been used to demonstrate that redundancy can be stably maintained over time (Nowak et al. 1997). However, the model requirement for perfect equivalency in gene functions and in mutations between paralogs seems unrealistic. Due to the challenges in assessing functions of paralogs, the extent of genetic redundancy and the factors contributing to it remain largely unclear.

Plants are an excellent resource for studying the fate of duplicated genes due to the relatively high rate of WGD events. Although pseudogenization (loss of gene function) is the most common fate of duplicated genes in plants (Panchy et al. 2016), some duplicates are retained. Duplicates may persist without selection for a few million years simply due to genetic drift (Panchy et al. 2016). In other cases, duplicates may be retained due to selection on novel, adaptive function through neo-functionalization (Ohno 1970) or mechanisms relevant to escape from adaptive conflict (Des Marais and Rausher 2008), and/or due to selection on existing functions through gene dosage increase (Ohno 1970), duplication degeneration complementation (i.e., subfunctionalization; Force et al. 1999), gene balance (Freeling and Thomas 2006), or paralog interference (Baker et al. 2013).

Beyond the mechanism of retention, by identifying and comparing characteristics of paralogous gene pairs and singleton genes, studies have revealed unique characteristics among retained duplicates. For example, there is a lower synonymous substitution rate among retained (i.e., not pseudogenized) paralogs derived from WGDs (Jiang et al. 2013), suggesting that these gene pairs are relatively recent duplicates or that there is selective pressure to retain the ancestral (or a more recently evolved) function. Retention bias is also seen for some gene functions. For example, paralogous transcription factor (TF) and signaling genes are retained at a higher rate than DNA repair genes (Blanc and Wolfe 2004). Retention rates of paralogs also vary by duplication mechanism—tandem duplicates (TDs) involved in stress responses are more frequently retained (Hanada et al. 2008), and genes involved in signaling processes are preferentially retained when derived from WGD rather than smaller duplication events (Maere et al. 2005). Although these studies reveal some characteristics of genes that are retained after duplication, they do not directly address whether these retained paralogs maintain redundant functions. A landmark study in Arabidopsis addressed this question using machine learning to integrate 43 gene features related to sequence similarity and gene expression, and predicted that approximately 50% genes in the Arabidopsis genome have at least one redundant paralog (Chen et al. 2010). In this study, a gene whose single mutant showed no abnormal phenotype (or a mild phenotype) and its closest match in the genome based on sequence similarity were defined as a redundant pair. The most important features for predicting redundancy included differences in isoelectric point, molecular weight, and predicted protein domains between genes in a pair. Although this pioneer study provided insights into the prevalence of genetic redundancy, redundancy was defined in only one way. Also, in the decade since that study substantially more functional genomic data have become available; inclusion of these data in addition to sequence similarity and gene expression may improve the accuracy of redundancy predictions.

Although the definition of redundancy presented above is prevalent, observation of unequal genetic redundancy, where the single mutant for one paralog shows a much more severe phenotype than the other and the double mutant (DM) has a still more severe phenotype (Briggs et al. 2006), promotes the idea that redundancy is more accurately conceptualized as a continuum. However, the time-consuming nature of precise phenotyping required to quantify redundancy in this manner means that such data are available for relatively few paralogs, and discussions of genetic redundancy frequently exclude single mutants with severe phenotypes. Here we build upon previous work by modeling genetic redundancy using multiple definitions of redundancy by including single mutants in multiple phenotypic categories, and incorporating over 4,000 gene features from six categories, including functional annotations, evolutionary properties, protein sequence properties, gene expression patterns, epigenetic modifications, and network properties. We compared several machine learning algorithms and feature selection methods to identify which of the features have the most predictive power with respect to redundancy. We additionally performed statistical analysis to identify features common among redundant gene pairs using nonredundant gene pairs as a contrast. To estimate the prevalence of genetic redundancy throughout the genome, we used two of the best-performing genetic redundancy definitions (RDs) to predict whether approximately 18,000 gene pairs in the Arabidopsis genome are genetically redundant. Finally, to assess the accuracy of our model, we validated predictions using a “holdout” testing data set and a handful of experimentally well-characterized gene pairs.

## Results and Discussion

### Definitions of Genetic Redundancy

**Fig. 1.**
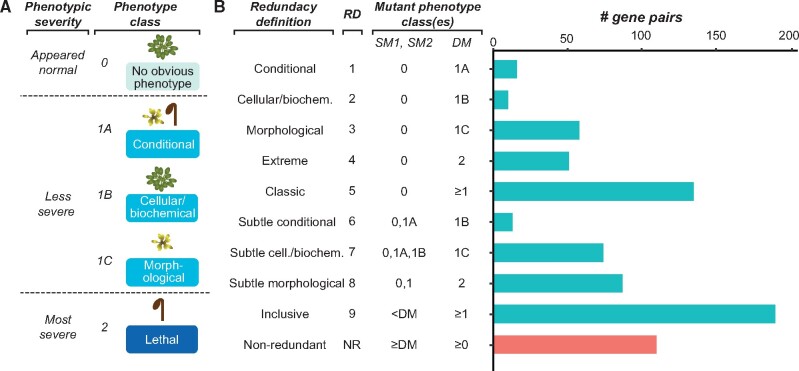
(*A*) Phenotype severity classification from Lloyd and Meinke (2012) and our corresponding phenotype classes. (*B*) Definitions of redundancy and NR based on phenotype classes of both single mutants (SM1 and SM2) and the DM for each gene pair. Descriptive definition names as well as a definition number and the number of gene pairs assigned to the definition are shown for each. RD5 (classic redundancy) is RD1-4 combined and RD9 (inclusive redundancy) is RD1-8 combined.

The designation of a gene pair as genetically redundant requires phenotype data for DMs and the corresponding single mutants. To define a set of benchmark redundant and nonredundant gene pairs, we used phenotype data for 2,400 single and 347 higher-order Arabidopsis mutants (including 271 DMs) from a previous study (Lloyd and Meinke 2012) in which mutants were classified as having no phenotype, a less severe phenotype (i.e., conditional, cellular/biochemical, or morphological), or a severe phenotype (i.e., lethal, indicating the gene is essential) based on comparison with wild-type individuals. We assigned these categories phenotype class numbers: 0 (no phenotype), 1A (conditional), 1B (cellular/biochemical), 1C (morphological), and 2 (lethal) (fig. 1*A*) and applied this same phenotype classification to 29 additional gene pairs (Bolle et al. 2013), resulting in a final benchmark set of 300 single and DM trios (two single mutants and one corresponding DM). Note that our data are from experiments generally not designed to assess genetic redundancy and typically conducted in one or a limited number of conditions and environments. Thus, it is more straightforward to identify an abnormal phenotype in a single mutant (i.e., phenotype distinct from wildtype, indicative of nonredundancy [NR]) than to prove the absolute absence of an abnormal phenotype (indicative of redundancy).

Using the benchmark phenotype data and the core idea for defining genetic redundancy based on comparison of the phenotype severity between single mutants and the corresponding DM, we established nine RDs (fig. 1*B*). These were intended to capture the heterogeneity in how genetic redundancy is viewed and defined, accounting for several different ways of thinking about what constitutes genetic redundancy and allowing us to examine less-studied types of redundancy (e.g., where a single mutant has a severe phenotype, or where a DM has a relatively mild phenotype): 1) clear and extreme examples of genetic redundancy, where single mutants have no apparent abnormal phenotype and the DM is lethal (RD4); 2) classic genetic redundancy, where single mutants have no abnormal phenotype and the DM has any of a range of phenotype severities (RDs 1–5); 3) subtle genetic redundancy, where single mutants have an abnormal phenotype that may be only slightly less severe than that of the DM (RDs 6–8); and 4) inclusive genetic redundancy, which encompasses all of the above in a single definition (RD9). Under our inclusive genetic RD, 190 of the gene pairs in our data set were classified as redundant.

This use of multiple definitions offered insulation against errors due to the inherent challenges of classifying phenotypes into specific categories. For example, some morphological phenotypes are much more severe than others; under specific conditions, conditional lethal is effectively the same as lethal). Another example is that, although RD4 (extreme redundancy) excluded DMs with conditional phenotypes (phenotype class 1A), both lethal and conditional lethal were included in the classic redundancy and inclusive RDs. Although we acknowledge that this classification of phenotype severity has caveats, in the absence of quantitative phenotype data on a large scale, qualitative categories together with our multiple definitions of redundancy allow us to better utilize the data set and begin addressing redundancy more as a continuum than as a binary problem.

To define nonredundant gene pairs, a single definition was used: two genes were considered nonredundant if the DM was in the same phenotype class as either single mutant or in a class with a lower number; that is, at least one single mutant had an equal or more severe phenotype than the DM (fig. 1*B*). The nonredundant set contained 110 gene pairs. The nearly 2:1 ratio of redundant to nonredundant gene pairs may reflect a bias in the literature. In the case of single mutants, plants are generally examined for phenotypes in large-scale screens in standard growth chamber conditions where they are not challenged, potentially masking conditional phenotypes. This would give the false impression that many single mutants have no abnormal phenotype, implying they are redundant. In the case of DMs, the presence of a more severe phenotype would tend to be reported, with negative results less likely to appear in the literature. Because comparably fewer gene pairs for which the DM has no abnormal phenotype have been reported, our data set likely contains comparably fewer nonredundant gene pairs (and conversely more redundant gene pairs) than there are in nature. DMs with much more dramatic phenotypes compared with the single mutants were also overrepresented in our data set (supplementary fig. S1, Supplementary Material online), likely for similar reasons. As a result, some definitions that included only DMs with mild or no phenotypes had too few gene pairs (RDs 1, 2, and 6, which had 16, 10, and 13 gene pairs, respectively) to generate robust models and were therefore excluded from further analyses.

### Optimal Parameters for Prediction of Genetic Redundancy with Machine Learning

**Fig. 2.**
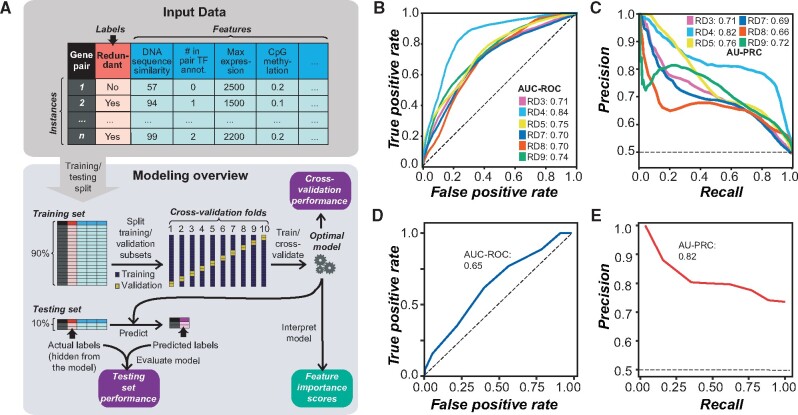
(*A*) Machine learning pipeline workflow. Input data consisted of instances (gene pairs) with labels (redundant or nonredundant) and values of features (characteristics of gene pairs). Example features, as shown in the table, include DNA sequence similarity, the number of genes in a pair annotated as having TF activity, maximum gene expression level, and the average level of CpG methylation among genes in the pair. The full input data are provided in supplementary data, Supplementary Material online. Instances were first split into training and testing sets. The training set was further split into a training subset (90%) and validation subset (10%) in a 10-fold cross-validation scheme. The optimal model after tuning the model parameters was used to provide performance metrics based on cross-validation, predict labels in the testing set for model evaluation purposes, and to obtain feature importance scores. (*B*, *C*) Cross-validation performance of models built using six of the nine RDs based on (*B*) AUC-ROC and (*C*) AU-PRC for each RD. RD1, 2, and 6 were not included due to small training data sizes. A model classifying gene pairs perfectly would have AUC-ROC and AU-PRC scores of 1.0; black dotted lines represent the performance of a model classifying at random, in which AUC-ROC and AU-PRC scores would be 0.5 given that we used balanced data (i.e., equal number of redundant and nonredundant instances). These curves represent the average scores from 100 iterations of model building; curves including standard deviation from this process are shown in supplementary figure S3, Supplementary Material online. (*D*) AUC-ROC and (*E*) AU-PRC for a model trained using extreme redundancy (RD4) gene pairs and balanced nonredundant pairs was applied to inclusive redundancy (RD9) gene pairs (excluding RD4) and nonredundant pairs that did not overlap with those used in training the RD4 model.

Machine learning allows integration of multiple data types to build a statistical model that can predict a specific outcome. In our case, we were interested in establishing a machine learning model that could predict whether a gene pair was redundant or not using six broad categories of data: functional annotations, evolutionary properties, protein properties, gene expression patterns, epigenetic modifications, and network properties (supplementary table S1, Supplementary Material online). The general approach we took is illustrated in figure 2*A*. Here the input for the model consisted of benchmark gene pairs (instances), classified as redundant or nonredundant (labels) according to our nine definitions, and information about the genes and gene pairs from the six categories of data (referred to as features). Performance was measured using the area under the curve-receiver operating characteristic (AUC-ROC); higher scores indicate a higher true positive rate (proportion of all redundant gene pairs correctly predicted) over the range of false positive rates (proportion of gene pairs incorrectly predicted as redundant). Performance was additionally measured using the area under the precision–recall curve (AU-PRC); higher scores here indicate greater precision (proportion of gene pair predictions that are correct) over the range of true positive rates (“recall”). Because we used a binary classification scheme (redundant or not) for machine learning, a model classifying gene pairs at random would have a score of 0.5 for both the AUC-ROC and AU-PRC measures, whereas a perfect model would have a score of 1. Comparing three commonly used machine learning algorithms, we determined that Support Vector Machines (SVM) performed the best on our data (see Materials and Methods and supplementary fig. S2*A* and *B*, Supplementary Material online). Thus, only models built using SVM are discussed in the following sections.

We next explored how the number of features examined and feature value transformation affected model performance. Although models using multiple features generally perform better than those based on single features, the presence of uninformative features can decrease model performance. Therefore, comparing two algorithms for feature selection, we tested model performance with different numbers of features. Additionally, we looked at the effect of transformation because transforming feature values (e.g., taking the square of values) can amplify small differences, allowing subtle patterns to be more readily identified. Using the inclusive RD (RD9), we tested 24 feature combinations (see Materials and Methods and supplementary table S2, Supplementary Material online) by asking how well the model based on each feature combination performed in predicting the benchmark gene pairs in a cross-validation scheme. We found that using 200 features selected with Random Forest, using the best transformations of each, led to the best performing model (AUC-ROC = 0.74, supplementary fig. S2*C*, Supplementary Material online, and AU-PRC = 0.72, supplementary fig. S2*D*, Supplementary Material online), with a 15% and 18% improvement in performance over a model using all of the untransformed features (AUC-ROC = 0.64, supplementary fig. S2*E*, Supplementary Material online, and AU-PRC = 0.61, supplementary fig. S2*F*, Supplementary Material online).

The selected features included many that were different representations of the same, raw feature. For example, several features related to total synonymous substitution rate (*Ks*), namely maximum *Ks*, minimum *Ks*, average *Ks*, difference in *Ks*, and total (sum) *Ks* for genes in a pair (see Materials and Methods) were all among the features selected for the inclusive redundancy model, demonstrating that representing a characteristic such as *Ks* in a variety of ways provides distinct and useful information for building the model. Including multiple representations and transformations of some features as described above explicitly introduced collinearity among features as a potentially confounding factor; collinearity likely already existed in our data set among different but related features, for example, duplication event and *Ks*. To determine whether this presented an issue for model performance (Dormann et al. 2013), we used principal component analysis (PCA) for the inclusive redundancy model to generate a new set of ten features based on the top ten PCs (explaining 53.4% of the total variance) from the selected 200 features. This model performed poorly (AUC-ROC = 0.65 and AU-PRC = 0.63), demonstrating that, although the PCA approach controls for collinearity, the resulting model is underfitted (even after inclusion of a total of 20 PCs explaining 69.8% of the variance: AUC-ROC = 0.70 and AU-PRC = 0.67).

### Comparison of Models Built with Different Redundancy Definitions

We anticipated that the training sets established using some RDs would result in more accurate predictions than others. Therefore, we next identified the RD that resulted in the best predictions of redundancy using the optimal algorithm (SVM) and input feature set that we identified (200 features selected with Random Forest, using only the best transformation of each feature). When comparing how well each model performed on the cross-validation sets, the model built using the extreme RD (RD4; trained model referred to as the extreme redundancy model) had the best performance (AUC-ROC = 0.84, fig. 2*B* and supplementary fig. S3*A*, Supplementary Material online; AU-PRC = 0.82, fig. 2*C* and supplementary fig. S3*B*, Supplementary Material online; light blue lines). This RD had the highest contrast between phenotypes of the single mutants (phenotype class 0: no apparent phenotype) and DMs (class 2: lethal). A likely reason for the better performance of the extreme redundancy model is that it was more straightforward to build a model to distinguish between redundant and nonredundant gene pairs when the phenotype differences were the most extreme. The second-best models were the ones with the largest training sample sizes, that is, classic redundancy (RD5) and inclusive redundancy (RD9; yellow and green lines, respectively, fig. 2*B* and *C* and supplementary fig. S3, Supplementary Material online). Thus, it appears that phenotype class contrast and sample size were the most important factors influencing model performance. We therefore focused on models built with the highest phenotype class contrast (extreme redundancy) and the largest sample sizes (classic redundancy and inclusive redundancy) for further model building.

Although the extreme redundancy model performed the best in cross-validation, the majority of redundant gene pairs in the Arabidopsis genome do not have such a high phenotype class contrast. We therefore tested whether the extreme redundancy model would prove useful in predicting redundancy between gene pairs when there were less extreme phenotype differences between the single and DMs. The extreme redundancy model was applied to a test set composed of inclusive redundancy gene pairs (after removing extreme redundancy pairs) and balanced nonredundant gene pairs. Although the AUC-ROC was only 0.65 (fig. 2*D*), the high AU-PRC score (0.82, fig. 2*E*) indicated that, as expected from applying a model built with a more conservative definition of redundancy, this model errs on the side of having a higher number of false negatives rather than false positives. We also applied the extreme redundancy model to the RD3, RD5, RD7, and RD8 data sets and the result is summarized in supplementary table S3, Supplementary Material online; in several cases, the performance of the extreme redundancy model on these definitions was comparable to or better than the performance of the definitions in cross-validation. Similarly, the classic redundancy model (RD5) was applied to a test set composed of inclusive redundancy gene pairs (after removing classic redundancy pairs) and balanced nonredundant gene pairs. The performance of this model on the inclusive redundancy gene pairs was significantly worse (AUC-ROC = 0.57, supplementary fig. S2*G*, Supplementary Material online; AU-PRC = 0.59, supplementary fig. S2*H*, Supplementary Material online) than the performance of the extreme redundancy model. Taken together, the best-performing models for predicting redundancy among gene pairs with all types of phenotype contrasts were those trained using the extreme redundancy and the inclusive RDs, but the extreme redundancy model can better predict redundancy based on other definitions. Therefore, these two models were used in the following analyses.

### Important Evolutionary Features in Predicting Redundant and Nonredundant Gene Pairs

**Fig. 3.**
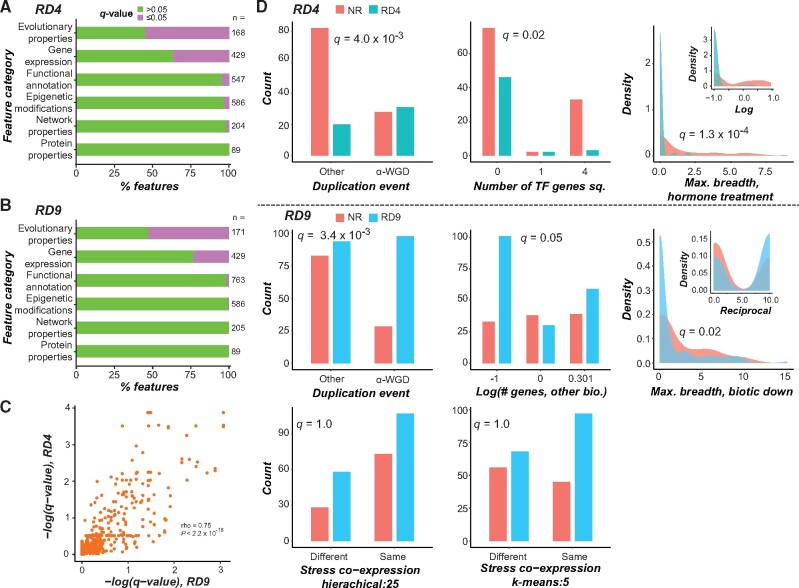
(*A*, *B*) Percentage of features in each feature category that were significantly associated with redundancy (Wilcoxon rank-sum test for continuous features; Fisher’s exact test for binary features; all multiple-test corrected with Benjamini–Hochberg method) when using (*A*) the extreme RD (RD4) and (*B*) the inclusive RD (RD9). (*C*) Correlation between RD4 and RD9 −log(*q*-values) obtained using the statistical tests as described in (*A*) and (*B*) for each feature. (*D*) Distribution of values among redundant and nonredundant gene pairs for selected features using the extreme redundancy and inclusive RDs (separated by a dotted line). For each model, a feature is shown here if the importance score ranked between 1 and 20 and it was the highest ranked in its feature category; *q*-values from the statistical tests described in (*A*) and (*B*) are inset in each graph. For transformed continuous features, untransformed feature values are shown, with transformed values shown as inserts. In the cases shown here, the *q*-values were the same for transformed and untransformed features. Abbreviations: “Number of TF genes sq.” is the square of the number of genes in the pair with the annotation DNA-dependent transcription factor; “Max. breadth, hormone treatment” is the maximum number of hormone treatments in which a gene in the pair is differentially expressed. “# genes, other bio.” is the number of genes in a pair with the GO annotation “other biological function.” “Max. breadth, biotic down” is the maximum number of genes in a pair downregulated under biotic stress. “Stress coexpression, hierarchical: 25” and “Stress coexpression, k-means: 5” refer to coexpression clusters generated from stress data sets with hierarchical (split into 25 clusters) and *k*-means (*k *=* *5) clustering, respectively; plots indicate the number of gene pairs in our data set for which genes in a pair are in the same cluster.

Because the identification of features that are distinct between redundant and nonredundant gene pairs can provide insights about the biological underpinnings of redundancy, we next assessed whether the distribution of values for each feature among the six feature categories was significantly different between redundant and nonredundant gene pairs (i.e., statistically associated with redundancy) based on the extreme redundancy and inclusive RDs (see Materials and Methods). For both extreme redundancy and inclusive redundancy, evolutionary properties had the highest percentage of features statistically associated with redundancy (55% and 53% respectively, multiple testing-adjusted *P-*value [*q*] *<*0.05; fig. 3*A* and *B*), and evolutionary property features tended to be the most significantly correlated with redundancy (median *q*-value of significant features = 3.0 × 10^−4^ and 4.0 × 10^−3^ respectively; supplementary fig. S4*A* and *B*, Supplementary Material online). Overall, a shared set of 159 features were significantly associated with redundancy in models trained with both the extreme and inclusive RDs, and there was a correlation between −log(*q*-values) for each feature in the extreme and inclusive redundancy models (Spearman’s rank *ρ* = 0.75, *P *<* *2.2 × 10^−^^16^; fig. 3*C*). This suggested that some features may be significantly associated with redundancy regardless of definition. However, among the top 200 features selected for building the extreme and inclusive models, we found that only 33% and 25%, respectively, were significantly associated with redundancy when considered individually (supplementary fig. S4*C* and *D*, Supplementary Material online), highlighting the utility of considering features jointly using machine learning.

We next looked into individual features that distinguished redundant gene pairs defined using extreme redundancy and inclusive redundancy from nonredundant gene pairs using feature importance scores output from the trained models (supplementary table S4, Supplementary Material online). In this case, an importance score represents the degree to which an individual feature contributes to the separation of redundant from nonredundant gene pairs by the algorithm, with features with a higher importance score having a larger contribution (see Materials and Methods). In total, 51 features were shared between the two models (supplementary table S4, Supplementary Material online) with well correlated importance ranks (Pearson’s correlation coefficient [PCC] = 0.63, supplementary fig. S5*A*, Supplementary Material online), suggesting that a core set of features are important for predicting redundancy using multiple definitions. However, a shared set of 51 features leaves approximately 75% of the 200 features selected for each model as unique, highlighting the significant effect of RD on the models and the types of important features recovered.

The relative importance of the six feature categories—ranked from best to worst based on median importance ranks for features in those categories in extreme redundancy/inclusive redundancy-based models—was as follows: functional annotations (32/17), evolutionary properties (63.5/81.5), network properties (123/81.5), gene expression patterns (110.5/101.5), epigenetic modifications (108/140), and protein properties (139/133.5). Note that the importance ranks do not mirror the findings in figure 3*A* and *B*, indicating that, for example, although the distributions of >50% of evolutionary property-based features significantly differed between redundant and nonredundant pairs, these features were not as important in predicting redundancy as functional annotation features. At first glance it may seem paradoxical that features significantly different between redundant and nonredundant gene pairs were not ranked as important by the model. However, this may occur when the difference is significant but the effect size is too small to reliably distinguish between the classes. The most important feature in both the extreme redundancy and the inclusive redundancy models, as determined by feature importance scores, was whether the gene pairs were duplicates from the ɑ-WGD event (for the importance scores of the top 20 features, see supplementary fig. S5*B* and *C*, Supplementary Material online), with ɑ-WGD-derived gene pairs more likely to be redundant (fig. 3*D*). The ɑ event is the most recent WGD event in the Arabidopsis lineage, and despite it having likely occurred approximately 50 Ma, the importance of this feature suggests that gene pairs derived from this event have not diverged in sequence and function sufficiently to appear nonredundant.

Two other evolutionary property features that were important for both definitions were whether two genes are reciprocal best matches (rank = 7 and 15 for extreme redundancy and inclusive redundancy, respectively, supplementary fig. S5*B* and *C*, Supplementary Material online) and a lethality score-derived feature (discussed below). Reciprocal best matches are paralogous gene pairs that do not have additional retained paralogs generated since their divergence; gene pairs that were reciprocal best matches were more likely to be redundant. As a pair of genes without more recent duplicates are themselves likely to be the product of a relatively recent duplication event (supplementary fig. S5*D*, Supplementary Material online), they are expected to have had less time to diverge in sequence and function, explaining their enrichment among redundant gene pairs. Consistent with this, *Ka-Ks*-related features ranked as high as 30 and 32 in the extreme and inclusive redundancy models, respectively. Nonetheless, contrary to our expectations, these evolutionary rate*-*related features were not the most informative. Instead, other characteristics confounded with rates of evolution, such as mechanism/mode of duplication and, as discussed in the following sections, gene functions and expression profiles, played more important roles in the model.

The difference in lethality score was an important feature in both models (reciprocal lethality score, defined as the reciprocal of the difference in lethality score between genes in a pair, rank = 2 and 9 for extreme and inclusive redundancy, respectively, supplementary fig. S5*B* and *C*, Supplementary Material online). Lethality score is the likelihood that mutation of a gene will lead to a lethal phenotype in Arabidopsis (Lloyd et al. 2015). Thus, we would expect that each gene in a redundant pair would have a low lethality score, and therefore a relatively small difference in lethality score for the gene pair. In contrast to our expectation, we found that redundant gene pairs generally had a smaller difference in reciprocal lethality scores (which equates to a larger difference in raw lethality score) compared with nonredundant gene pairs, although the difference between redundant and nonredundant gene pairs was not significant (Wilcoxon test, *q-*value < 0.11). This unexpected result was likely an artifact of a bias in our data—lethality scores were predicted by Lloyd et al. (2015) for genes without known single mutant phenotypes, but 92% of the genes included in our benchmark data set have known (nonlethal) phenotypes. In the absence of a predicted lethality score, we used a score of 0 for known nonlethal mutants, which likely artificially lowered the average lethality scores in our benchmark set. To determine whether the use of lethality score skewed the results, we ran the inclusive redundancy model with the lethality score-associated features excluded and found an insignificant difference in model performance: the model without lethality score-associated features had an AUC-ROC = 0.74 and AU-ROC = 0.73. We posit that the insignificant difference in model performance, despite the highly ranked importance of lethality score, is likely due to the presence of collinear features that would provide similar information.

### Important Gene Expression, Functional, and Network Features

Features related to gene expression made up the largest portion of features selected for extreme and inclusive redundancy model building, with a total of 126 gene expression features selected for one or both models. The predicted directionality of four features varied between the two definitions, meaning that for a given feature, redundant gene pairs according to one RD had higher values compared with nonredundant gene pairs, whereas the reverse was true for the other definition. For example, expression variation in the developmental expression data set (after transforming average values reciprocally) was higher for redundant gene pairs according to the extreme RD than for nonredundant gene pairs, but lower for redundant gene pairs according to the inclusive RD. We also found that tissue-specific stress responses varied by RD; the mean rank of features related to abiotic stress response for extreme redundancy was higher for root tissue (97) than shoot tissue (120), whereas the opposite was true for inclusive redundancy (99 and 94, respectively). Features derived from the developmental data set were not consistently informative across definitions; although there were four developmental gene expression features in the top 30 for inclusive redundancy, no such features ranked higher than 54 for extreme redundancy. The most important gene expression feature for inclusive redundancy was the maximum number of biotic stress conditions under which one or both genes in a pair was downregulated, with redundant gene pairs having a lower maximum than nonredundant gene pairs (fig. 3*D*). Thus, redundant gene pairs tend not to be downregulated under stress conditions. This is consistent with previous findings indicating that duplicate genes involved in stress responses are retained at a higher rate than genes involved in other processes (Maere et al. 2005). The most important gene expression feature for extreme redundancy was the maximum number of hormone treatments under which one or both genes in a pair was differentially expressed compared with the control, with nonredundant gene pairs having a higher maximum (fig. 3*D*).

Among 2,627 functional annotation features, 19 and 13 were among the top 200 for the extreme redundancy and inclusive redundancy models, respectively. Although only one of these features was selected for both models, given that functional enrichment among redundant gene pairs varies by RD (supplementary fig. S6, Supplementary Material online), it was expected that different functional annotation features would be important for predicting redundancy using different RDs. The most important gene function feature for the extreme redundancy model was the number of genes in a pair (0, 1, or 2) annotated as DNA-dependent TFs (referred to as TFs). In the trained extreme redundancy model, gene pairs in which both genes had this annotation were more frequently predicted as nonredundant, consistent with the feature value distributions (fig. 3*D*). This was somewhat unexpected as previous studies have shown that TFs are more likely to be retained after gene duplication than other types of genes (Blanc and Wolfe 2004). The most important functional annotation feature for the inclusive redundancy model was the number of genes in the pair having the annotation “other biological processes” (fig. 3*D*). This term, which encompasses a broad range of processes including responses to stressors or hormones, ion transport, circadian rhythm, aging, and cell growth, among many others, was an important predictor of nonredundant gene pairs.

Finally, although no network properties or protein properties were among the 20 most important features in predicting extreme redundancy, two network property features were in the top 20 important features for inclusive redundancy: presence in the same gene coexpression clusters, with gene pairs in the same cluster more likely to be redundant (fig. 3*D*). Consistent with this, Chen et al. (2010) found that gene coexpression during pathogen infection was one of the most important features for predicting redundancy in Arabidopsis. In that study, isoelectric point, overlap in protein domain annotations, and sequence similarity were also among the features found to be important predictors of redundancy. Although these features were included in our model building based on extreme and inclusive redundancy, they ranked between 26 and 166 depending on the RD (supplementary table S4, Supplementary Material online). The minimal overlap in features found to be important in predicting redundancy is likely due to the difference in how redundant gene pairs were defined; in Chen et al., they were “paralogous genes whose single mutants show little or no phenotypic defects but whose double and higher order mutant combination, when available, show a significant phenotype.” Our extreme RD is more stringent, encompassing only gene pairs whose DMs are lethal. Our inclusive RD takes into account phenotype severity in the context of the single and corresponding DM trios; that is, we include gene pairs whose DMs have relatively mild phenotypes so long as the single mutant phenotypes are less severe.

**Fig. 4.**
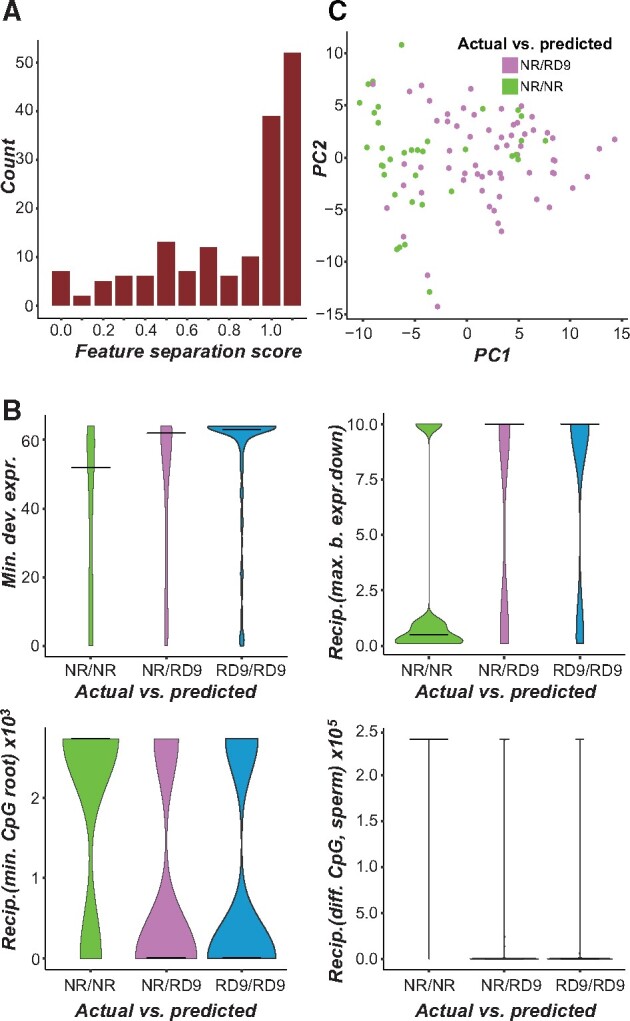
(*A*) Distribution of feature separation scores for features used to build the inclusive redundancy (RD9) model. To identify features that may contribute to mis-predictions, feature values were compared between 1) nonredundant gene pairs predicted as nonredundant (NR/NR), 2) nonredundant pairs predicted as redundant (NR/RD9), and 3) redundant pairs predicted as redundant (RD9/RD9). Redundant pairs predicted as nonredundant (RD9/NR) were not included in this analysis due to the small sample size. Using the median value (Med) in each class/predicted class category, we calculated a normalized feature separation score as follows: ${(\mathrm{Med}}_{\mathrm{NR}/\mathrm{RD}9\;}-\;{\mathrm{Med}}_{\mathrm{NR}/\mathrm{NR}})/({\mathrm{Med}}_{\mathrm{RD}9/\mathrm{RD}9}-{\mathrm{Med}}_{\mathrm{NR}/\mathrm{NR}})$. For each feature, the feature separation score represents the difference in feature values between correctly and incorrectly predicted nonredundant gene pairs, with a score of 0 meaning that correctly and incorrectly predicted pairs had the same feature values and a score of 1 meaning that incorrectly predicted pairs had the same feature values as redundant gene pairs. Close to 20% of the features had a separation score of 1. (*B*) Distribution of values for selected features among the three categories of actual and predicted redundancy described in (*A*). Horizontal bars indicate the median. “Min. dev. expr.” is the minimum number of tissues and developmental stages in which a gene in the pair is differentially expressed. “Recip. (max. b. expr. down)” is the reciprocal of the maximum number of biotic stress conditions in which one or both genes in the pair are downregulated. “Recip. (min. CpG root)” is the reciprocal of the minimum level of CpG methylation in root tissue for genes in the pair. “Recip. (diff. CpG sperm)” is the reciprocal of the difference in CpG methylation level in sperm cells for genes in the pair. These four features had a feature separation score close to 1 and had feature importance scores in the top 10 for the inclusive redundancy model, implicating them in mis-predictions. (*C*) Dimensions 1 and 2 of a PCA performed to identify features that were different between correctly and incorrectly predicted nonredundant pairs. Dimension 1 explains 18.1% of the variance and Dimension 2 explains 10.0% of the variance. The top 24 features contributing to Dimension 1 were related to CpG methylation levels (supplementary table S5, Supplementary Material online).

We also examined the potential causes of the mis-prediction of nonredundant gene pairs as redundant (the reverse case was rare and therefore not analyzed in detail) in the inclusive redundancy model, by comparing feature values between correctly and incorrectly predicted pairs and generating a score representing whether mis-predicted nonredundant pairs had feature values similar to inclusive redundancy pairs (fig. 4*A*, see Materials and Methods). We also identified features likely contributing to mis-predictions by considering the feature importance; although features with high importance scores generally aid in correct classification, they can contribute to mispredictions in specific cases. This is because features with high importance scores are weighted more heavily in generating predictions; therefore, if a nonredundant pair happens to have a value similar to those commonly seen in redundant gene pairs, the pair could be incorrectly predicted as redundant. We identified several features for which incorrectly predicted nonredundant pairs had values more like redundant gene pairs (using the inclusive RD) than correctly predicted nonredundant pairs, and that also had high feature importance scores, suggesting they may play a role in mis-predictions (fig. 4*B*). Additionally, in a PCA of correctly and incorrectly predicted nonredundant pairs (fig. 4*C*), the top 24 features contributing to the first principal component were related to CpG methylation (supplementary table S5, Supplementary Material online), implicating this type of methylation as a major contributor to mis-prediction.

Given the enrichment of some GO categories in gene pairs comprising the extreme redundancy and inclusive RDs (supplementary fig. S6, Supplementary Material online), one consideration is that our models may be biased toward features distinguishing genes in the enriched GO categories and thus are not generalizable to the whole genome, particularly to genes not in the enriched categories. To address this, we compared performance of the model on gene pairs in enriched and unenriched categories and found that there is no significant difference (supplementary table S6, Supplementary Material online). We therefore do not find evidence that any such enrichment in functions for our paralogs would lead to less accurate predictions on gene pairs without these annotations.

### Redundancy Predictions for Arabidopsis Gene Pairs Not in the Benchmark Data Set

With the predictive model of redundancy in place, we sought to answer two questions about genetic redundancy in Arabidopsis more broadly: 1) given the models, to what extent are paralogs in the genome redundant, and 2) whether paralogs derived from different duplication mechanisms and events differ in redundancy. As it was extremely computationally intensive to generate predictions for every paralogous gene pair in the Arabidopsis genome, we selected a subset of paralogous gene pairs to address these two questions: 1) all of the WGD and TD pairs in the Arabidopsis genome (7,764 total, collectively referred to as the WG/TD set; supplementary data, Supplementary Material online); 2) paralogs in a large gene family. The second data set was used because a gene family consists of a group of paralogs derived from a variety of duplication mechanisms and with differing evolutionary distances, it offers a wide spectrum of relatedness among gene pairs. For this analysis, we used the protein kinase (Kin) superfamily to generate all possible combinations of gene pairs, then randomly selected 10,000 pairs for analysis (supplementary data, Supplementary Material online). We expected that applying our model to both data sets would provide information about genetic redundancy at the genome-wide scale and at the more fine-grained gene family level. Although both the extreme and inclusive redundancy models showed high accuracy in predicting redundant gene pairs in cross-validation (87% and 92% of redundant gene pairs correctly predicted, respectively; supplementary fig. S7*A* and *B*, Supplementary Material online), the extreme redundancy model predicted nonredundant gene pairs with much higher accuracy than the inclusive redundancy model (75% and 36%, respectively; supplementary fig. S7*A* and *B*, Supplementary Material online). Because of the high error rate in predicting nonredundant pairs with the inclusive redundancy model, we focused on using the extreme redundancy model to estimate the prevalence of genetic redundancy in the Arabidopsis genome.

**Fig. 5.**
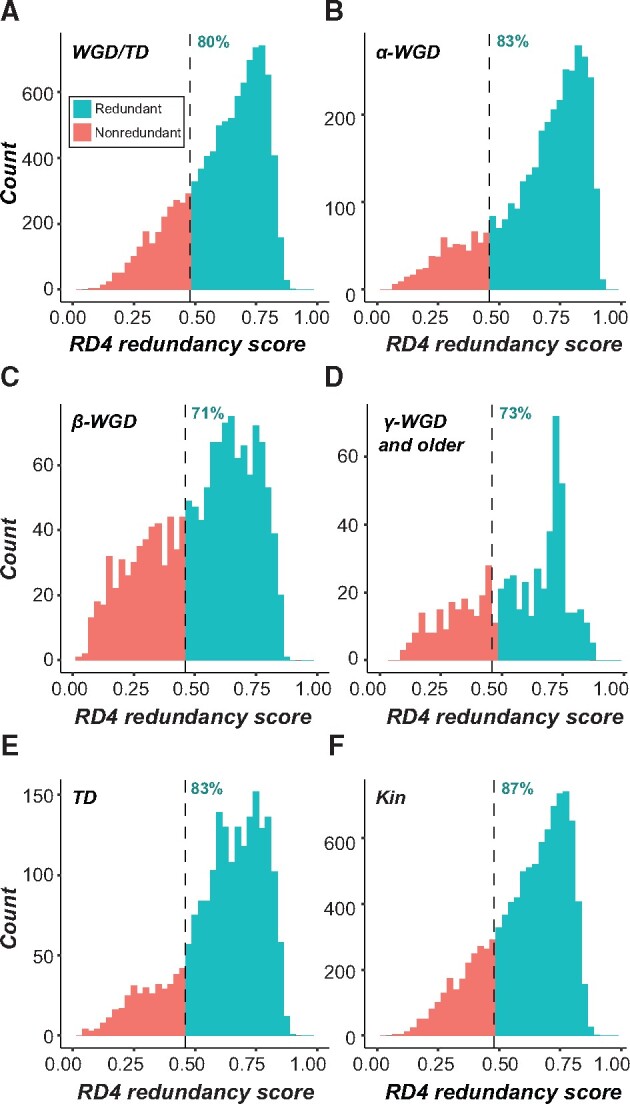
(*A*) Predicted redundancy scores from the extreme redundancy (RD4) model for gene pairs in the genome derived from whole genome or tandem duplication (WGD and TD, respectively). The results grouped specifically by duplication event/type are shown in (*B–E*): (*B*) Gene pairs derived from the α-WGD event, (*C*) gene pairs derived from the β-WGD event, (*D*) gene pairs derived from the γ-WGD event, (*E*) gene pairs derived from tandem duplication (TD). (*F*) Predicted redundancy scores of 10,000 randomly selected gene pairs from the kinase superfamily (Kin). A majority of gene pairs in all of these data sets were predicted as redundant using the extreme RD. Predictions as redundant or nonredundant are based on a threshold score selected within our machine learning pipeline to maximize F1 score, that is, the harmonic mean of precision (in this case, the proportion of true redundant pairs to predicted redundant pairs) and recall (proportion of redundant pairs predicted correctly), with gene pairs having a score above the threshold being called redundant and gene pairs with a score below the threshold being predicted as nonredundant.

Although we analyzed machine learning results primarily as a binary variable (gene pairs were classified as either redundant or nonredundant), these binary predictions were generated from likelihood scores output by the machine learning pipeline. The likelihood score, referred to as a “redundancy score,” ranges on a continuum from 0 to 1, with 0 being most likely nonredundant and 1 most likely redundant. Using this redundancy score, a threshold score was determined (as part of the machine learning pipeline) that would maximize the harmonic mean of precision (in this case, the proportion of true redundant pairs to predicted redundant pairs) and recall (proportion of redundant pairs predicted correctly), and this threshold was used to generate the binary predictions for the WG/TD and Kin data sets. Using the extreme redundancy model, the majority of the 17,764 WG/TD and Kin gene pairs were predicted as redundant with redundancy scores well above the threshold (fig. 5). Among the WG/TD set as a whole, 80% were predicted as redundant (fig. 5*A*), with gene pairs derived from the ɑ-WGD event more likely to be predicted as redundant (83%; fig. 5*B*) compared with those derived from the β-WGD event (71%; fig. 5*C*) and the γ and more ancient WGD events (73%, fig. 5*D*). As duplicate pairs evolve over time, it is expected that the degree of genetic redundancy would continue to decline. Although this is true when comparing the ɑ-WGD to older events, similar proportions of duplicate pairs from the β and more ancient events were predicted as redundant based on RD4. This may be because gene pairs derived from the more ancient γ-WGD look similar to those derived from the β-WGD in terms of *Ks* (Maere et al. 2005). However, it is surprising that so many seemingly redundant gene pairs (based on the extreme RD) that duplicated 50 Ma (ɑ-WGD), 80 Ma (β-WGD; Edger et al. 2015) or longer would be retained. Similarly, 83% of TDs and 87% of kinases were predicted as redundant based on the extreme RD (fig. 5*E* and *F*, respectively).

This percentage of redundant pair predictions was higher than previous estimates in the literature (e.g., Chen et al. 2010). It is important to note that in our WG/TD and Kin data sets, gene pairs are likely being predicted as redundant because they more closely resemble redundant gene pairs with respect to features that have the highest weight in our predictive model (e.g., WGD event). However, the model is built on experimental data that have much more power when calling a gene pair as nonredundant than calling them as redundant; demonstrating that mutants have a severe abnormal phenotype is simpler than definitively stating that a mutant has no abnormal phenotype. As previously proposed (Bouché and Bouchez 2001; Bolle et al. 2013), the lack of an observed severe phenotype in a single mutant may be because phenotypes are conditional, tissue specific, and/or subtle rather than masked by genetic redundancy. Many large-scale phenotyping studies are not able to take these factors into account, and it would therefore be expected that a model built with data from such studies overestimate genetic redundancy in the genome. This is reflected in our result showing that misclassifications by our model on the benchmark data set were overwhelmingly nonredundant pairs predicted as redundant, with very few instances of the reverse.

Although the binary classification of gene pairs as redundant or nonredundant was possible with the available data and straightforward to interpret, it is an over-simplification of the complex nature of genetic redundancy. The threshold-based definition of genetic redundancy may be convenient, but the landscape of genetic redundancy is far more nuanced—there are gene pairs with various degrees of genetic redundancy, not simply redundant or not. Nonetheless, these data still allowed us to gain valuable insights into the mechanistic underpinnings of genetic redundancy by revealing important features as discussed in the earlier sections. In addition, we anticipate the models can be iteratively improved with the future availability of more phenotype data, particularly quantitative data.

### Validation of Predictions

**Fig. 6.**
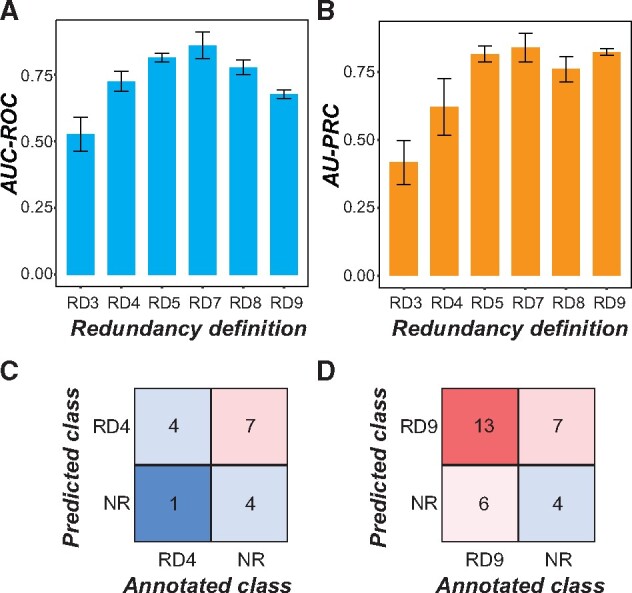
(*A*) AUC-ROC and (*B*) AU-PRC values for the holdout testing sets for models built with each RD. RDs 1, 2, and 6 were not included in the analysis due to small sample size. Performance of the models on testing sets was lower compared with performance in cross-validation (fig. 2*B* and *C* and supplementary fig. S3, Supplementary Material online), likely due to the small sizes of the testing sets. (*C*, *D*) Heat map of the confusion matrix for (*C*) extreme redundancy (RD4) and (*D*) inclusive redundancy (RD9) models showing the number of correctly and incorrectly predicted redundant and nonredundant gene pairs in the respective testing sets.

To validate predictions, we used a “holdout” testing set (10%, 16 and 30 pairs for RD4 and RD9, respectively, randomly selected and proportionally divided between redundant and nonredundant pairs, fig. 2*A*) of the benchmark data. This testing set was not included in the model building process and serves to illustrate how well the model will perform on new data. Applying the extreme and inclusive redundancy models on the testing sets for those definitions, we obtained AUC-ROC scores of 0.73 and 0.68, respectively (fig. 6*A*) and AU-PRC scores of 0.62 and 0.82, respectively (fig. 6*B*). Although there was a decrease in performance compared with cross-validation results (fig. 2*B* and *C*), 80% (4/5) and 68% (13/19) of redundant pairs were predicted correctly based on the extreme and inclusive redundancy models, respectively, and 36% (4/11) of nonredundant pairs were predicted correctly by each of these models (fig. 6*C* and *D*). Thus, the holdout testing set generally supported the utility of the extreme and inclusive redundancy models, but the current threshold score was more conservative toward calling gene pairs as nonredundant. The small sample size of the testing sets likely contributed to the decreased performance of the models compared with their performance in cross-validation, as bias in such a small sample could impact the results. However, due to the relatively small size of the benchmark data set as a whole, withholding more than 10% of gene pairs from the training step may have introduced bias to the trained models and therefore would not have been an efficient use of the available data.

Further validation was performed by identifying single and DMs in the literature that have specifically been studied as mutant trios and have very well documented and characterized phenotypes. We selected ten of these gene pairs: five that meet our criteria for redundancy under the inclusive definition and five we would classify as nonredundant (supplementary table S7, Supplementary Material online). Half of the pairs were present in our inclusive redundancy benchmark training data set, whereas the other half were present in the WG/TD and/or kinase test data sets. We examined the predictions of these known gene pairs from the literature in the cross-validation and testing sets, and found that the inclusive redundancy model correctly predicted four of five redundant pairs but mis-predicted all five of the nonredundant pairs as redundant. The predictions of the same gene pairs were also examined for the extreme redundancy model; however, three of the gene pairs defined as redundant using the inclusive definition could not be defined as redundant using the extreme RD because the DMs were not lethal. Thus, this testing set for the extreme redundancy model included only two redundant gene pairs. The extreme redundancy model correctly predicted one out of the two redundant pairs and four out of the five nonredundant pairs. This was consistent with our expectations and prior results showing that the inclusive redundancy model tends to err on the side of predicting false positives whereas the extreme redundancy model is much more conservative and prone to generating false negative predictions (supplementary table S8, Supplementary Material online).

To determine why mis-predictions may have occurred in these specific cases, we revisited features previously identified as likely contributors to mis-prediction in general in the benchmark data set (e.g., fig. 4*A* and *B*). For the inclusive redundancy (RD9) model, one such feature was reciprocal best match. Although this feature was more strongly associated with nonredundant gene pairs in the benchmark data set (supplementary fig. S8*A*, Supplementary Material online), the one redundant pair predicted by the RD9 model as nonredundant (RD9/nonredundant) comprised paralogs that were not reciprocal best matches, making this a likely reason for mis-prediction. Derivation of paralogs from the ɑ-WGD event was another such feature (supplementary fig. S8*B*, Supplementary Material online); three nonredundant pairs predicted as redundant (nonredundant/RD9) were derived from the ɑ-WGD event, indicating that this feature was a likely contributor to their mis-prediction. Another important feature was related to the number of biotic stress conditions under which genes were downregulated (referred to as biotic downregulation breadth). For this feature, the distribution of feature values among the actual/predicted classes demonstrated that all five nonredundant/RD9 pairs had values more similar to the correctly predicted RD9 pairs than to the correctly predicted nonredundant pairs (supplementary fig. S8*C*, Supplementary Material online). For the extreme redundancy (RD4) model, the one redundant pair that was predicted as nonredundant (RD4/nonredundant) had values for features related to CpG methylation (supplementary fig. S8*D*, Supplementary Material online), gene family size (supplementary fig. S8*E*, Supplementary Material online), and CHH methylation (supplementary fig. S8*F*, Supplementary Material online) that were more similar to those of nonredundant pairs. Additionally, all four of the nonredundant pairs predicted as redundant (nonredundant/RD4) had CHH methylation in embryo tissue values that were more similar to those of RD4 gene pairs (supplementary fig. S8*F*, Supplementary Material online).

In total, we identified several types of features that were likely contributors to mispredictions, including duplication event (ɑ-WGD or not), downregulation under biotic stress conditions, and gene methylation patterns. Importantly, we were thus able to identify one or more features that likely contributed to each instance of mis-prediction of both the extreme redundancy and the inclusive redundancy models on the gene pairs used for validation, an important step in improving future iterations of the model. For example, depending on the definition being used and the importance of the accuracy of predictions (precision) compared with the importance of identifying all redundant gene pairs in a data set (recall), certain features could be excluded from the model.

## Conclusions

In this study, we optimized and utilized a machine learning approach to predict genetic redundancy among paralogs in Arabidopsis using multiple definitions of redundancy. We identified two biologically relevant and well-performing definitions of redundancy and the optimal 200 features for each definition that allowed us to best model redundancy. Several features related to evolutionary properties, including lethality score, whether genes in a pair were reciprocal best matches, and the type of duplication event from which a gene pair was derived, were consistently ranked as important in generating predictions across RDs. Interestingly, evolutionary rates, such as *Ka* and *Ks*, were statistically different between redundant and nonredundant gene pairs but not highly ranked in the models, indicating that multiple factors contribute to redundancy, as revealed by machine learning models integrating multiple features. Analysis of these evolutionary-related features demonstrated that redundant gene pairs tend to be more recent duplicates than nonredundant pairs. Although it may be tempting to explain redundancy as gene pairs having not had enough time to diverge in function, many redundant pairs are derived from a WGD event estimated to have occurred approximately 50 Ma, offering plenty of time for pseudogenization. This suggests that there may be some selective pressure to maintain redundancy. In general, we found feature importance to be highly variable by RD, underscoring the need for testing multiple definitions depending on the biological question being addressed. For example, if one is interested in predicting which genes are lethal or have severe phenotypes a stricter definition is required than when a broader view of redundancy is being used, whereby less extreme phenotype contrasts between single and DMs would be appropriate.

Although the models provide useful information about gene features related to genetic redundancy, the models are far from perfect and there remains room for improvement in terms of prediction accuracy. Performance on testing gene pairs withheld from model building was generally not as good as model performance in cross-validation, which may be due to the unavoidably small size of the testing sets. In addition, our more conservative trained model predicted 84% of 17,764 paralogs throughout the genome to be redundant, which is a much higher estimate than has been shown previously (Chen et al. 2010). This is likely a result of the underlying data used for model building; our models are expected to be biased toward categorization of gene pairs as redundant for the following reasons. We classified redundancy using phenotype data from the literature, including experiments that were not specifically designed to identify redundancy; there are expected to be substantial differences between experiments in how phenotypes were scored. For example, conditional or particularly subtle phenotypes may not have been examined. This likely results in misclassification of single mutants as not having an abnormal phenotype. Because genetic redundancy was defined as a DM having a more severe phenotype than the corresponding single mutants, this bias will therefore lead to overestimation of genetic redundancy.

Furthermore, classification of gene pairs as redundant or nonredundant, as we were able to do using the broad phenotype categories currently available on a large scale, overly simplifies a complex phenomenon. Redundancy as it exists in nature is not an all-or-nothing binary state, but rather a continuum with a wide range of biologically relevant states. In our modeling exercise, redundancy scores derived from the model allow an approximation of this continuum, which can be further tested. One approach for testing the degree of genetic redundancy is by obtaining lifetime fitness data for single and DM sets. Because lifetime fitness in a mutant reflects the totality of phenotypic effects due to the introduced mutation over the entire life cycle of the individual, subtle, and conditional phenotypes are likely better captured. Importantly, our current model predicts redundancy as defined by differences in some phenotypes under some specific conditions. It remains unclear the extent to which such a model is relevant to predicting redundancy when it is defined based on single and DM fitness, the phenotypic outcome that has the most bearing on the evolutionary fate of a gene pair. Thus, in future studies the generation of lifetime fitness data would allow for a machine learning regression model that more accurately predicts degrees of genetic redundancy between genes in a pair rather than simply classifying genes as redundant or not. Such a model could be applied to gene pairs within a large gene family to compare predicted redundancy scores and reveal patterns related to redundancy maintenance and loss through evolutionary time. Analysis of features important for building the model would be expected to yield additional useful insights about mechanisms related to the evolutionary fate of gene duplicates and the long-term retention of genetic redundancy.

Despite these limitations, the prediction models can distinguish redundant and nonredundant genes as defined here with reasonable accuracies. In addition, we view this work as an initial step in an ongoing effort to accurately model genetic redundancy that provides a framework for future modeling, in which better phenotype data can be included. Taken together, our results demonstrate the utility of machine learning in combining features to generate accurate predictions of genetic redundancy and identify several evolutionary features that are important in predicting genetic redundancy across several definitions.

## Materials and Methods

### Definitions of Redundant and Nonredundant Gene Pairs

Arabidopsis mutant phenotype data were collected from Lloyd and Meinke (2012) and Bolle et al. (2013). Our benchmark data set comprised mutant trios for which a DM phenotype and both corresponding single mutant phenotypes were reported, with a total of 300 mutant trios. A numeric phenotypic severity value was assigned to each single and DM (fig. 1*A*), with 0 representing no abnormal phenotype; 1A, a conditional phenotype of any kind; 1B, a cell or biochemical phenotype; 1C, a morphological phenotype; and 2, a lethal phenotype. Redundancy was classified using nine RDs of varying stringency (fig. 1*B*). The least stringent definition was inclusive redundancy (RD9), in which any gene pair for which the DM phenotype severity score was higher than that of both the single mutants was defined as redundant. With this definition, the data set contained 190 redundant gene pairs. Gene pairs were classified as nonredundant if at least one single mutant had a phenotype severity score greater than or equal to the DM score; the data set contained 110 nonredundant gene pairs.

### Feature Value Generation

For predictive modeling, data from six general categories were collected for each gene: functional annotations such as GO terms; evolutionary properties such as synonymous substitution rate; protein sequence properties such as posttranslational modifications; gene expression patterns; epigenetic modifications such as histone methylation; and network properties such as gene interactions based on functional gene network data (supplementary table S1, Supplementary Material online). These data were processed to generate feature values for each gene pair (supplementary data, Supplementary Material online), and the method used for processing depended on the data type: binary (e.g., whether or not a gene had a given protein domain), categorical (e.g., all the names of protein domains present in a given gene product) or continuous (e.g., gene expression level).

Features such as protein domain and functional annotations were treated as binary and/or categorical input data for feature generation. For processing as binary input data, each gene was assigned a value of 0 (does not have the annotation/property) or 1 (has the annotation/property); gene pair feature values were then generated by taking the number of genes in the pair (0, 1, or 2) having that annotation/property. For example, if Gene1 was annotated as having DNA-binding activity but Gene2 was not, the feature value for DNA-binding activity for that gene pair would be 1. Additional features were generated by taking the square, log_10_, and reciprocal value of features processed in this way. For processing as categorical input data, all annotations of a specific type (e.g., GOslim terms) were listed for each gene. These were then used to represent similarity between genes in a pair. For example, if Gene1 had functional annotations of “DNA-binding activity” and “signal transduction” and Gene2 had functional annotations of “signal transduction” and “protein binding,” the number of overlapping annotations would be 1, the total number of unique annotations between the gene pair would be 3, and the percent overlap would be 33. For continuous data, gene pair feature values were generated by calculating the difference, average, maximum, minimum, and total of the values for the gene pair. For example, if Gene1 had an isoelectric point of 10 and Gene2 had an isoelectric point of 9, the difference would be 1, the average 9.5, the maximum 10, the minimum 9, and the total value would be 19. Additional features were generated by taking the square, log_10_, and reciprocal of features processed as categorical and continuous data, and by assigning each value to one of four quartile bins generated from the untransformed feature data. Additionally, PCA was conducted using all transformed and untransformed feature data, and the top five components included as features.

### Functional Annotation and Evolutionary Property Features

Functional annotations included GO biological process, molecular function and cellular component annotations (Ashburner et al. 2000; Gene Ontology Consortium 2017), metabolic pathway annotations from AraCyc v.15 (Mueller et al. 2003), and predicted protein domain annotations from Pfam (Finn et al. 2016). These annotations were processed as binary and categorical data as described above. There were 2,627 features related to functional annotations after transformations were applied (supplementary table S1, Supplementary Material online).

Broadly, evolutionary properties included duplication mechanism and timing, and relationship to other genes in the genome. There were 171 features related to evolutionary properties after transformations were applied (supplementary table S1, Supplementary Material online).

To get the evolutionary rate for each gene in a pair, protein sequences (collected from NCBI; Pruitt et al. 2007) of each *A. thaliana* gene pair were searched against protein sequences from *Theobroma cacao*, *Populus trichocarpa*, *Glycine max*, and *Solanum lycopersicum*, using the Basic Local Alignment Search Tool for protein sequences (BlastP; Altschul et al. 1990). Protein sequences of the gene pair and the best hits in these four species were first aligned using MUSCLE (Edgar 2004), and then were compared with their coding nucleotide sequences to generate the corresponding coding sequence (CDS) alignment. CDS alignments were used to build gene trees using RAxML/8.0.6 (Stamatakis 2014) with parameters: -f a -x 12345 -p 12345 -# 1000 -m PROTGAMMAJTT. *Ka*, *Ks*, and the *Ka*/*Ks* ratio on branches leading to each gene of a gene pair were calculated using the free-ratio model of the codeml program in PAML v. 4.9d (Yang 2007). Gene family size and lethality scores were obtained from Lloyd et al. (2015). Where lethality scores were not available, a score of 0 was assigned to known nonlethal genes and 1 was assigned to known lethal genes. Nucleotide and amino acid sequence similarity were calculated using EMBOSS Needle (McWilliam et al. 2013). *Ka*, *Ks*, *Ka*/*Ks*, gene family size, functional likelihood, lethality scores, and sequence similarity were processed as continuous data.

Gene pairs were determined to have been derived from one of four types of gene duplication events using MCScanX-transposed (Wang et al. 2013): 1) segmental duplicates—paralogs located in corresponding intraspecies collinear blocks; 2) TDs—paralogs next to each other; 3) proximal duplicates—paralogs close to each other, but separated by ≤10 nonhomologous genes; 4) transposed duplicates—one of the paralogs located in inter-species collinear blocks, the other not. Segmental duplicates were additionally noted as being derived or not derived from the α- or β-WGD events. Protein sequences of *A. thaliana* were searched against protein sequences of *A. thaliana* (intra-species), *Arabidopsis lyrata*, *Brassica rapa*, *Carica papaya*, *P. trichocarpa*, and *Vitis vinifera* (interspecies) using BlastP, with a cutoff *E*-value of 1 × 10^−10^. Five different sets of parameters were evaluated for MCScanX-transposed: 1) -k 50 -s 5 -m 25, 2) -k 50 -s 2 -m 25, 3) -k 25 -s 2 -m 25, 4) -k 25 -s 2 -m 50, and 5) -k 25 -s 5 -m 25; where -k indicates the cutoff score of collinear blocks, -s specifies the number of matched genes required for the calling of a collinear block, and -m means the maximum number of genes allowed for the gap between two genes. The duplication mechanisms inferred using these five different sets of parameters were consistent with one another for the majority of gene pairs; 78 pairs had discrepant results, representing 0.4% of the total data set. In these cases, the mechanism that occurred most frequently in the results for that gene pair was assigned; if there was no majority, the mechanism was listed as N/A. Each gene pair was assigned a binary value indicating whether or not the genes were reciprocal best matches (i.e., they were one another’s best hit based on nucleotide Blast searches) and whether or not they were derived from each type of duplication mechanism (e.g., a gene pair derived from the α-WGD event would have a value of 1 for the WGD feature and for the α-WGD feature, and a value of 0 for all other duplication mechanisms).

Retention rate was based on the presence or absence of a paralog in 15 species: *A. lyrata*, *Capsella rubella*, *B. rapa*, *T. cacao*, *P. trichocarpa*, *Medicago truncatula*, *V. vinifera*, *S. lycopersicum*, *Aquilegia coerulea*, *Oryza sativa*, *Amborella trichopoda*, *Picea abies*, *Selaginella moellendorffii*, *Physcomitrella patens*, and *Marchantia polymorpha*. The retention rate for each gene was calculated as the number of genomes in which a paralog was present divided by the total number of genomes analyzed (16: *A. thaliana* plus the 15 additional species). Genome data were collected from Phytozome (Goodstein et al. 2012) for *P. patens* 318 v3.3, *M. polymorpha* 320 v3.1, *S. moellendorffii* 91 v1.0, *A. trichopoda* 291 v1.0, *O. sativa* 323 v7.0, *B. rapa* 277 v1.3, *C. rubella* 183 v1.0, *A. thaliana* 167 TAIR10, *A. lyrata* v2.1, *M. truncatula* 285 Mt4.0 v1, *V. vinifera* 145 Genoscope 12x, *A. coerulea* v3.1, *P. trichocarpa* 210 v3.0, and *T. cacao* 233 v1.1; from NCBI for *S. lycopersicum* v2.5; and from PlantGenIE (Sundell et al. 2015) for *P. abies* v1.0.

### Gene Expression and Epigenetic Modification Features

Processed microarray gene expression data sets were obtained from Moore et al. (2019) and contained gene expression levels under biotic (Wilson et al. 2012) and abiotic stress (Kilian et al. 2007; Wilson et al. 2012), under hormone treatment (Goda et al. 2008), at different developmental stages (Schmid et al. 2005), and at different times of day (Mockler et al. 2007). In addition to these gene expression levels, we also considered expression breadth, which represents the number of tissues and conditions under which each gene is expressed. Gene expression levels and ribosome occupancy from RNA-seq and Ribo-Seq experiments in root tissue were obtained from Hsu et al. (2016) and processed along with the microarray gene expression data as continuous data. There were 450 features related to gene expression after transformations were applied (supplementary table S1, Supplementary Material online).

Epigenetic modifications included DNA methylation, chromatin accessibility, and histone modifications. Percent CHH, CHG, and CpG methylation, gene body methylation, and histone modification data were obtained from Lloyd et al. (2015). Percent methylation values were treated as continuous data, and gene body methylation and histone modification data as binary data. Chromatin accessibility data were from Sullivan et al. (2014) and were treated as binary features, with each gene receiving a value of 1 if it contained a DNase peak site and a value of 0 if it did not. There were 565 features related to epigenetic modifications after transformations were applied (supplementary table S1, Supplementary Material online).

### Protein Sequence and Network Property Features

Protein sequence properties included amino acid length, isoelectric point, and posttranslational modifications. Amino acid lengths were obtained from Lloyd et al. (2015). Isoelectric points and myristoylation data were from The Arabidopsis Information Resource (Berardini et al. 2015). Amino acid length and isoelectric point were processed as continuous data. Acetylation, deamination, formylation, hydroxylation, oxidation, and propionylation data were obtained from The Plant Proteome Database (Sun et al. 2009). Posttranslational modifications were processed as binary data: whether or not the protein product was predicted or known to have the modification. In total, 93 features were related to protein sequence properties after transformations were applied (supplementary table S1, Supplementary Material online).

Network properties were related to known or potential interactions of genes or protein products. Gene interactions based on functional gene network data (AraNet, Lee et al. 2010) and protein–protein interactions (AtPIN, Brandão et al. 2009) were processed as categorical data. Gene coexpression-related features were calculated from the microarray data sets referenced above using multiple clustering algorithms, namely *k*-means, c-means and hierarchical clustering at *k *=* *5, 10, 25, 50, 100, 200, 300, 400, 500, 1,000, and 2,000 as described in Moore et al. (2019). These data were processed as categorical data, with each combination of clustering algorithm, data set and *k*-value included as a feature; a gene pair received a value of 1 if both genes were in the same cluster and a value of 0 if they were not. There were 205 features related to network properties after transformations were applied (supplementary table S1, Supplementary Material online).

### Identification of Features Distinguishing Redundant and Nonredundant Pairs

To identify features that could distinguish between gene pairs from the redundant and nonredundant classes, we applied statistical tests to determine if feature values were significantly different between the classes. Binary gene pair features (e.g., duplication type, presence in a gene coexpression cluster) were analyzed using two-sided Fisher’s exact tests with multiple testing correction using the Benjamini–Hochberg method (Benjamini and Hochberg 1995). To determine whether feature value transformations improved the ability to distinguish between classes, the reciprocal, square, and log_10_ of continuous features were included as separate features. Continuous values were also binned into four quartiles of equal size and bin values were included as features. Transformed and untransformed continuous feature values between redundant and nonredundant gene pairs were analyzed using a Wilcoxon rank sum test (Wilcoxon 1945) with multiple test correction performed using the Benjamini–Hochberg method. Features were considered to be able to distinguish between redundant and nonredundant gene pairs if *q *<* *0.05 after multiple testing correction (supplementary table S1, Supplementary Material online). Continuous feature effect sizes are the standardized *z* statistic (calculated from the *P* values given by the Wilcoxon rank sum test) divided by the square root of the sample size. Binary feature effect sizes correspond to the odds ratio calculated from the enrichment table for each feature.

### Redundancy Prediction Model Building and Optimization with Machine Learning

Models for predicting genetic redundancy between gene pairs were built with Random Forest, Gradient Boosting, and SVM algorithms implemented in the scikit-learn machine learning package (Pedregosa et al. 2011) in Python; scripts used for model building are available at https://github.com/ShiuLab/Manuscript_Code/tree/master/2021_Arabidopsis_redundancy_modeling. Before establishing any model, 10% of the benchmark data set was held out as the test data set, which was used to evaluate the performance of the final models. The remaining 90% of the data set was used to establish the models. To balance the numbers of redundant and nonredundant gene pairs when building the model, nonredundant gene pairs were randomly down-sampled to the same number as that of redundant gene pairs, and this down-sampling was repeated 100 times to prevent any potential sample bias in the models, resulting in 100 balanced data sets. For Random Forest and Gradient Boosting, a grid search was performed with 10-fold cross-validation for parameter optimization: redundant and nonredundant gene pairs in a balanced data set were randomly and proportionally divided into ten folds, nine of which were used to train the model (training set, 90%) and one was used to evaluate the model performance (validation set, 10%). This scheme was repeated ten times to ensure that each of the ten folds were used as the validation set once, thus ten models were built and the average performance for ten validation sets was reported; this 10-fold cross-validation scheme was conducted for the first ten balanced data, and hyperparameters with the highest average cross-validation performance were selected to build the final models using the 100 balanced data sets. Hyperparameters optimized were learning rate for Gradient Boosting; maximum depth and maximum features for Gradient Boosting and Random Forest; number of trees (“N estimators”) for Random Forest; and C parameter for SVM. Hyperparameter values used for models discussed in this study are shown in supplementary table S9, Supplementary Material online. Performance in cross-validation was also used to set a threshold score for the trained model in calling gene pairs as redundant or nonredundant. Thresholds are selected within our machine learning pipeline to maximize F1 score, that is, the harmonic mean of precision (in this case, the proportion of true redundant pairs to predicted redundant pairs) and recall (proportion of redundant pairs predicted correctly), for a total of 100 models. The 10-fold cross-validation was also used when building those 100 models.

Parameters tested for optimal model performance were the machine learning algorithm, the number of features included in the model, the feature selection algorithm, and the type of data transformations used. We first compared the performance of Gradient Boosting, Random Forest, and SVM using different numbers of features from one to 4116 (the full feature set). SVM was on average the best-performing algorithm when using the inclusive redundancy model (RD9, supplementary figure S2*A* and *B*, Supplementary Material online; ANOVA, *P*-value < 2 × 10^−^^16^, and Tukey’s Honestly Significant Difference test, *q* values < 0.008). Further optimization consisted of identifying the number of features to be included in the final model (narrowed down in the previous step to 50, 100, 200, or 500), the algorithm with which those features should be selected (Random Forest or Elastic Net [EN]), and whether data transformation improved model performance (log_10_, square, reciprocal of each value, and binning as described above). Specifically, we tested the effect on model performance of using only features with no transformations applied (“NT”), allowing multiple transformations of the same original feature to be included (“MT”), or the best transformation for each feature (as determined by feature importance scores from the trained models; “BT”). Twenty-four models varying these parameters were tested (supplementary fig. S2*C* and *D*, Supplementary Material online). The optimal combination was 200 features selected with Random Forest and only the best transformation of a feature allowed; these parameters were used in further model building. A comparison of the optimal feature combination with the 200 features selected with EN when the best transformation of a feature was allowed is in supplementary table S10, Supplementary Material online.

Models trained on the extreme redundancy and inclusive redundancy data sets were used to determine features that were important in predicting gene pairs as redundant or nonredundant. When SVM is performed with a linear kernel and normalized feature values, the model-learned weights associated with each feature can be used to determine feature importance. The greater the absolute magnitude of the feature weight, the more important that feature in the model’s predictions. We used the absolute value of the feature weights output by the model to determine feature importance. The performance of the model on new data was evaluated using the testing data set (10% held out from model building as described above).

Some models were trained using one definition of redundancy and applied to a data set of a different definition, for example, applying the trained extreme redundancy model to the inclusive redundancy data set. In this case, the training set consisted of the extreme redundancy gene pairs and a randomly selected half of the nonredundant gene pairs; the test set to which the model was applied consisted of the other half of the nonredundant gene pairs and the redundant gene pairs in the inclusive redundancy data set that were nonoverlapping with the gene pairs in the extreme redundancy data set. This process was the same for all models where the training and testing sets used different definitions of redundancy.

The trained extreme redundancy and inclusive redundancy models were used to predict redundancy among all tandem and WGD pairs in Arabidopsis (supplementary data, Supplementary Material online) and among a random sample of Arabidopsis kinase gene pairs. Using kinase family classifications from Lehti-Shiu and Shiu (2012), all possible within-family combinations of gene pairs were generated. Ten thousand of these pairs were then randomly selected for predictions (supplemental data, Supplementary Material online).

## Supplementary Material

Supplementary data are available at *Molecular Biology and Evolution* online.

## Supplementary Material

msab111_Supplementary_Data
