# Supplementary material for: Predictive Models of Genetic Redundancy in Arabidopsis thaliana
Source: Mol Biol Evol. 2021 Apr 19;38(8):3397–414. doi: 10.1093/molbev/msab111 (PMC8321531; doi:10.1093/molbev/msab111)
Supplement: msab111_Supplementary_Data [file msab111_supplementary_data.zip › Supplemental_figures_revision.pdf]

Fig. S1. Distribution of benchmark gene pairs among phenotype severity categories (as defined in **Figure 1**) for both single mutants (SM1 and SM2) and the double mutant for each pair. The dataset is biased toward double mutants with more severe phenotypes.

Fig. S2. (*A*) AUC-ROC scores and (*B*) AU-PRC scores for binary classification machine learning models built using RD9 with Gradient Boosting (GB), Random Forest (RF) and Support Vector Machine (SVM) algorithms and using different numbers of features. Shading indicates the standard deviation from 100 iterations of the model using balanced datasets (see **Methods**). Using AUC-ROC and AU-PRC as a measure, models built with SVM generally performed the best. (*C*) AUC-ROC scores and (*D*) AU-PRC scores for machine learning models built using different combinations of feature numbers ("# Feat."), feature selection algorithms ("FS alg."), and numbers of transformations allowed for each feature ("Trans."; "MT" = multiple transformations of a feature allowed; "BT" = best transformation of a feature allowed; "NT" = no transformed features used; see **Methods** for details). Different letters indicate statistically significant differences between models. Using AUC-ROC as a measure, the best-performing combination was 200 features selected with Random Forest and with only the best transformation of each feature allowed. Using AU-PRC as a measure, this combination was significantly better than all other combinations of parameters (ANOVA, $p < 2 \times 10^{-16}$; Tukey's HSD, p-values $< 2.3 \times 10^{-4}$) except for the following two combinations: 200 features selected with Random Forest, with multiple transformations of each feature allowed, and 100 features selected with Random Forest, with multiple transformations of each feature allowed (Tukey's HSD, p-values 1.00 and 0.13, respectively). (*E*) AUC-ROC curve and (*F*) AU-PRC curve of a model built with all untransformed features, demonstrating the improved performance of the optimized model in (*C*) and (*D*) with respect to both measures. (*G*) AUC-ROC and (*H*) AU-PRC for a model trained using classic redundancy gene pairs (RD5) and balanced nonredundant pairs then applied to inclusive redundancy gene pairs (RD9 gene pairs excluding RD5) and nonredundant pairs that did not overlap with those used in training the classic redundancy model. Using these performance measures, this model did not perform as well as the model trained using the extreme redundancy definition (RD4) when applied to a test set composed of RD9 gene pairs and balanced nonredundant pairs as described (**Figure 2D-E**); therefore, the classic redundancy definition was not selected for further analysis.

Fig. S3. Cross-validation performance of models built using six of the nine redundancy definitions based on (*A*) AUC-ROC and (*B*) AU-PRC for each redundancy definition. These are the curves shown in **Figure 2B-C**, here with shading indicating the standard deviation from 100 iterations of model building. RDs 1, 2, and 6 were not included due to small training data sizes. A model classifying gene pairs

perfectly would have AUC-ROC and AU-PRC scores of 1.0; black dotted lines represent the performance of a model classifying at random, in which AUC-ROC and AU-PRC scores would be 0.5 given that we used balanced data (i.e., equal number of redundant and nonredundant instances).

Fig. S4. (*A*-*B*) Distribution of -log(*q*-values) from tests of feature association with redundancy as defined using (*A*) extreme redundancy (RD4) and (*B*) inclusive redundancy (RD9). Statistical significance was determined with Wilcoxon rank sum test for continuous features and two-sided Fisher's exact test for binary features; all values were corrected for multiple testing with the Benjamini-Hochberg method. The dotted lines show a *q*-value of 0.05. All *p*-values, *q*-values and effect sizes are reported in **Table S1**. The median effect sizes (calculated as described in **Methods**) were 0.11 (RD4) and 0.07 (RD9) among continuous features, and 1.4 (RD4) and 1.1 (RD9) among binary features. (*C*-*D*) Distribution by feature category of the 200 features selected for model building for (*C*) the extreme redundancy and (*D*) the inclusive redundancy definitions. Features that have a statistically significant association with redundancy as described above are shown in orange. Only 25% of the features selected were significantly associated with redundancy.

Fig. S5. (*A*) Comparison of feature importance ranks of the 51 features included in both the extreme redundancy (RD4) and inclusive redundancy (RD9) models. The feature importance ranks are well correlated between the two models (PCC = 0.63; $p = 6.0 \times 10^{-7}$), indicating that a core set of features is important in predicting redundancy across definitions. (*B*-*C*) Raw feature importance scores obtained from machine learning models for (*B*) the extreme redundancy definition and (*C*) the inclusive redundancy definition. Raw feature importance scores vary between models and cannot be directly compared; normalized feature importance scores for direct comparison between models are shown in **Table S4**. (*D*) Distribution of *Ks* values among gene pairs included in the extreme redundancy (left) and inclusive redundancy (right) models that are reciprocal best matches (orange) and not reciprocal best matches (green). Dotted lines show the median *Ks* value for each group. Gene pairs that are reciprocal best matches tend to be more recent duplicates as shown by the lower *Ks* values.

Fig. S6. Enrichment of GO terms among redundant gene pairs vs. nonredundant gene pairs for each redundancy definition. Blue represents enrichment among nonredundant pairs while red represents enrichment among redundant gene pairs; lighter shades show statistically weaker associations (i.e., higher *q*-value) and darker shades show statistically stronger associations (lower *q*-value). Statistically significant enrichment was seen in transcription factor activity among nonredundant pairs compared with RD4 and RD8 gene pairs, and in DNA-dependent transcription factor activity among nonredundant gene

pairs compared with RD4, RD5 and RD9 gene pairs. In general, functional enrichment varied highly by the definition used.

Fig. S7. (*A*-*B*) Performance in cross-validation; the percentages of (*A*) redundant and (*B*) nonredundant gene pairs correctly and incorrectly predicted using different redundancy definitions are shown. Gene pairs to the left of the threshold selected in the machine learning pipeline (dotted line; see **Methods**) were classified as nonredundant, and gene pairs to the right were classified as redundant. Models were built using extreme redundancy (RD4) pairs, inclusive redundancy (RD9) pairs, and inclusive redundancy pairs not included in the extreme redundancy definition as the redundant instances (RD9 minus RD4) with balanced nonredundant pairs.

Fig. S8. (*A*) Distribution of reciprocal best match gene pairs (RBM) among annotated (Ann.) vs. predicted (Pred.) classes using the inclusive redundancy definition (RD9): true negatives (NR/NR), false negatives (RD9/NR), false positives (NR/RD9), and true positives (RD9/RD9), including the benchmark dataset and the 10 validation pairs identified from the literature (here referred to as validation pairs). Two NR/RD9 validation pairs were reciprocal best matches, which was observed more often for RD9/RD9 pairs than NR/NR pairs, while genes in the RD9/NR validation pair were not reciprocal best matches, likely explaining these three mis-predictions. (*B*) Distribution of α-whole genome duplication (α-WGD)-derived gene pairs among the annotated/predicted classes, including the benchmark and 10 validation pairs. Three validation NR/RD9 pairs were derived from the α-WGD event, which was observed more often for RD9/RD9 pairs than NR/NR pairs, potentially contributing to their mis-prediction. (*C*) Distribution of feature values among benchmark and validation gene pairs for the maximum biotic downregulation breadth between genes in a pair; a reciprocal transformation was applied to generate reciprocal maximum biotic downregulation breadth. All five of the validation NR/RD9 pairs had high values for this feature and looked more similar to RD9/RD9 pairs than to NR/NR pairs. (*D*) Distribution of reciprocal minimum CpG methylation in endosperm cells values among benchmark and validation pairs, using the extreme redundancy definition (RD4). (*E*) Distribution of total gene family size values among benchmark and validation pairs. The RD4/NR validation pair had a high value, which was more consistent with the values of NR/NR pairs than RD4/RD4 pairs. (F) Distribution of reciprocal average CHH methylation in embryo tissue values among benchmark and validation pairs. All four of the NR/RD4 validation pairs had high values that were more similar to those of RD4/RD4 gene pairs, while the one RD4/NR pair had a low value more similar to those of NR/NR pairs.

Figure S1

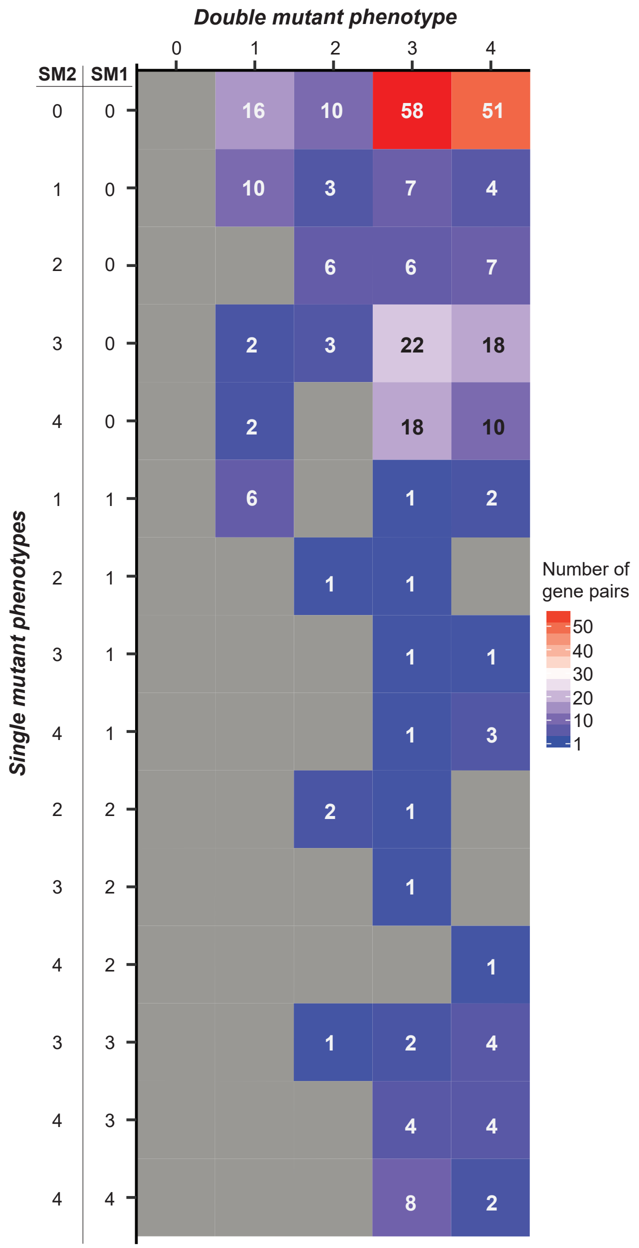

Double mutant phenotype
0
1
2
3
4
SM2
SM1
Single mutant phenotypes
16
10
58
51
10
3
7
4
6
6
7
2
3
22
18
2
18
10
6
1
2
1
1
1
1
1
3
2
1
1
1
1
2
4
4
4
8
2
Number of gene pairs
50
40
30
20
10
1

Figure S2

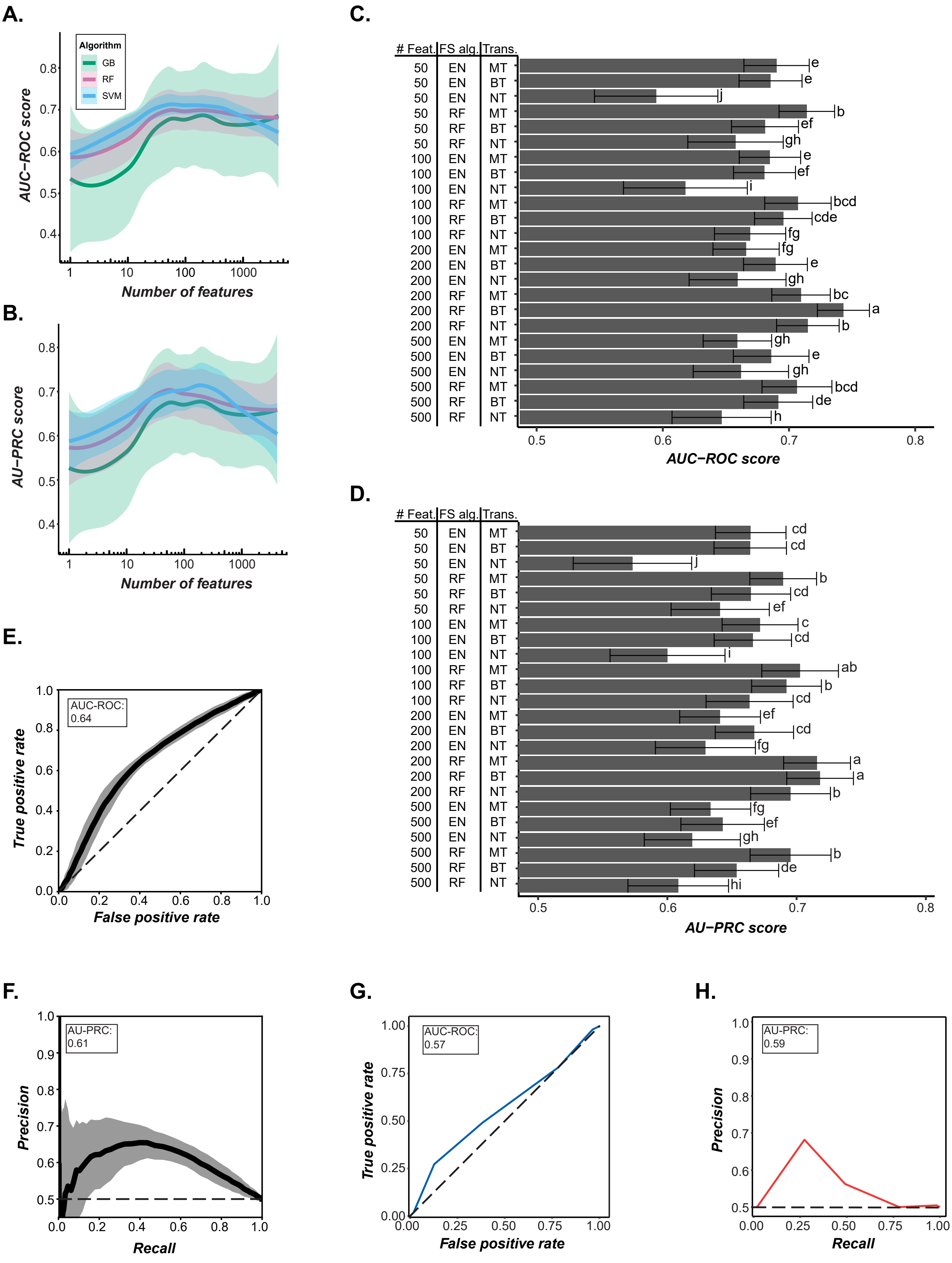

A.
Algorithm
GB
RF
SVM
AUC−ROC score
Number of features
B.
AU−PRC score
Number of features
C.
Feat.
FS alg.
Trans.
AUC−ROC score
D.
Feat.
FS alg.
Trans.
AU−PRC score
E.
AUC-ROC:
0.64
True positive rate
False positive rate
F.
AU-PRC:
0.61
Precision
Recall
G.
AUC-ROC:
0.57
True positive rate
False positive rate
H.
AU-PRC:
0.59
Precision
Recall

Figure S3

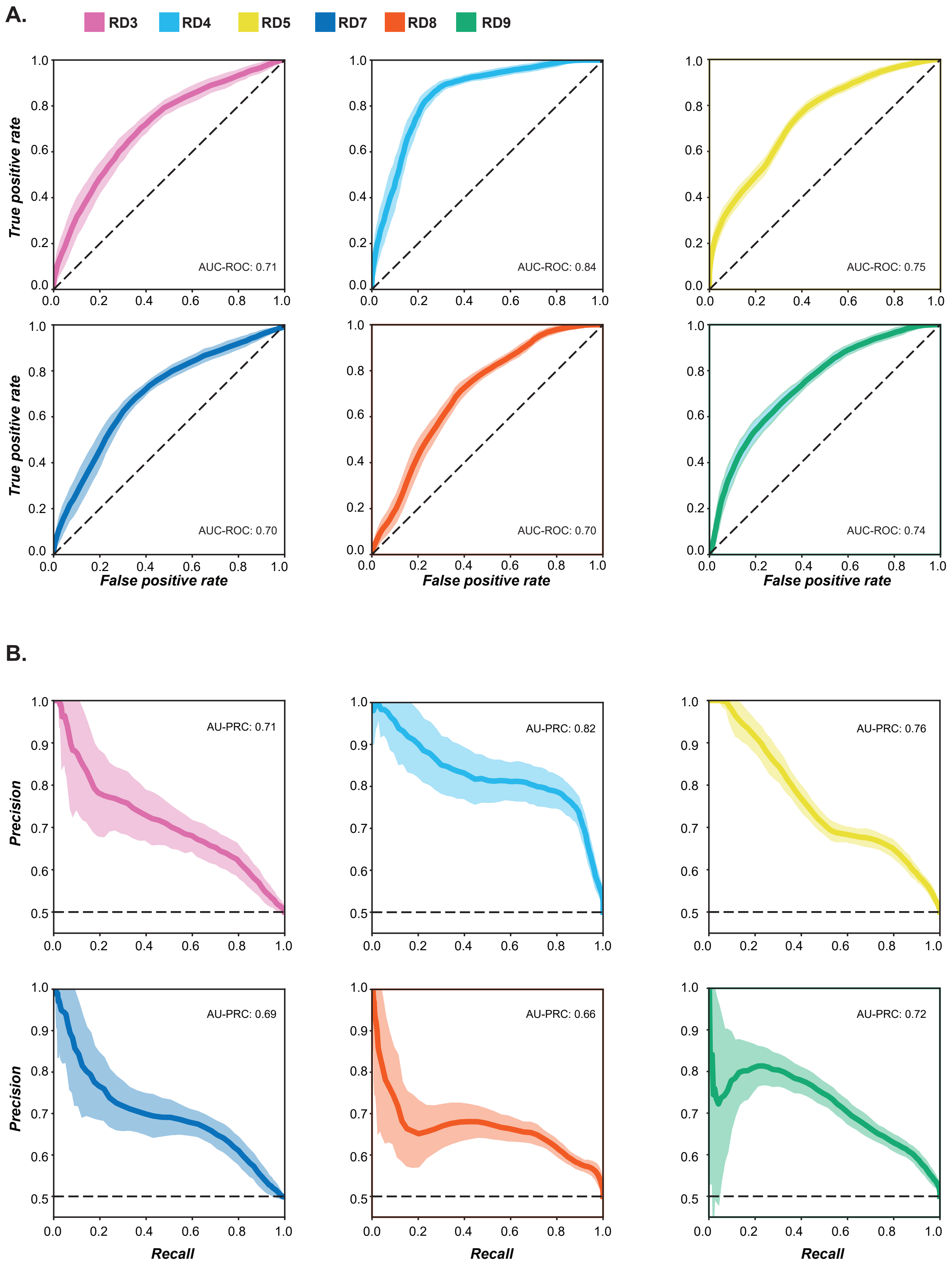
A.
RD3
RD4
RD5
RD7
RD8
RD9
True positive rate
False positive rate
AUC-ROC: 0.71
AUC-ROC: 0.84
AUC-ROC: 0.75
AUC-ROC: 0.70
AUC-ROC: 0.70
AUC-ROC: 0.74
B.
Precision
Recall
AU-PRC: 0.71
AU-PRC: 0.82
AU-PRC: 0.76
AU-PRC: 0.69
AU-PRC: 0.66
AU-PRC: 0.72

Figure S4

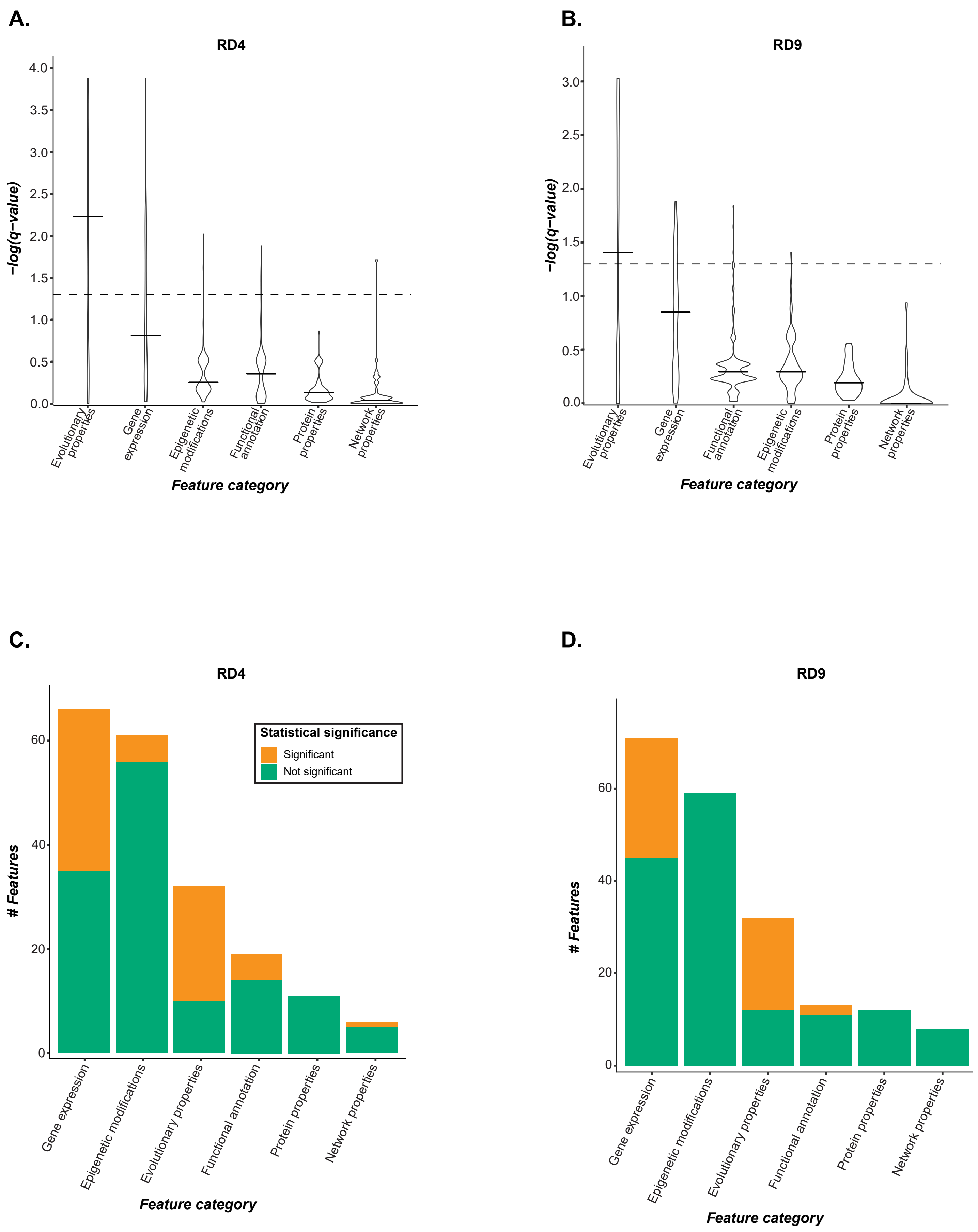
A.
RD4
−log(q−value)
4.0
3.5
3.0
2.5
2.0
1.5
1.0
0.5
0.0
Evolutionary properties
Gene expression
Epigenetic modifications
Functional annotation
Protein properties
Network properties
Feature category
B.
RD9
−log(q−value)
3.0
2.5
2.0
1.5
1.0
0.5
0.0
Evolutionary properties
Gene expression
Functional annotation
Epigenetic modifications
Protein properties
Network properties
Feature category
C.
RD4
Features
60
40
20
0
Statistical significance
Significant
Not significant
Gene expression
Epigenetic modifications
Evolutionary properties
Functional annotation
Protein properties
Network properties
Feature category
D.
RD9
Features
60
40
20
0
Gene expression
Epigenetic modifications
Evolutionary properties
Functional annotation
Protein properties
Network properties
Feature category

Figure S5

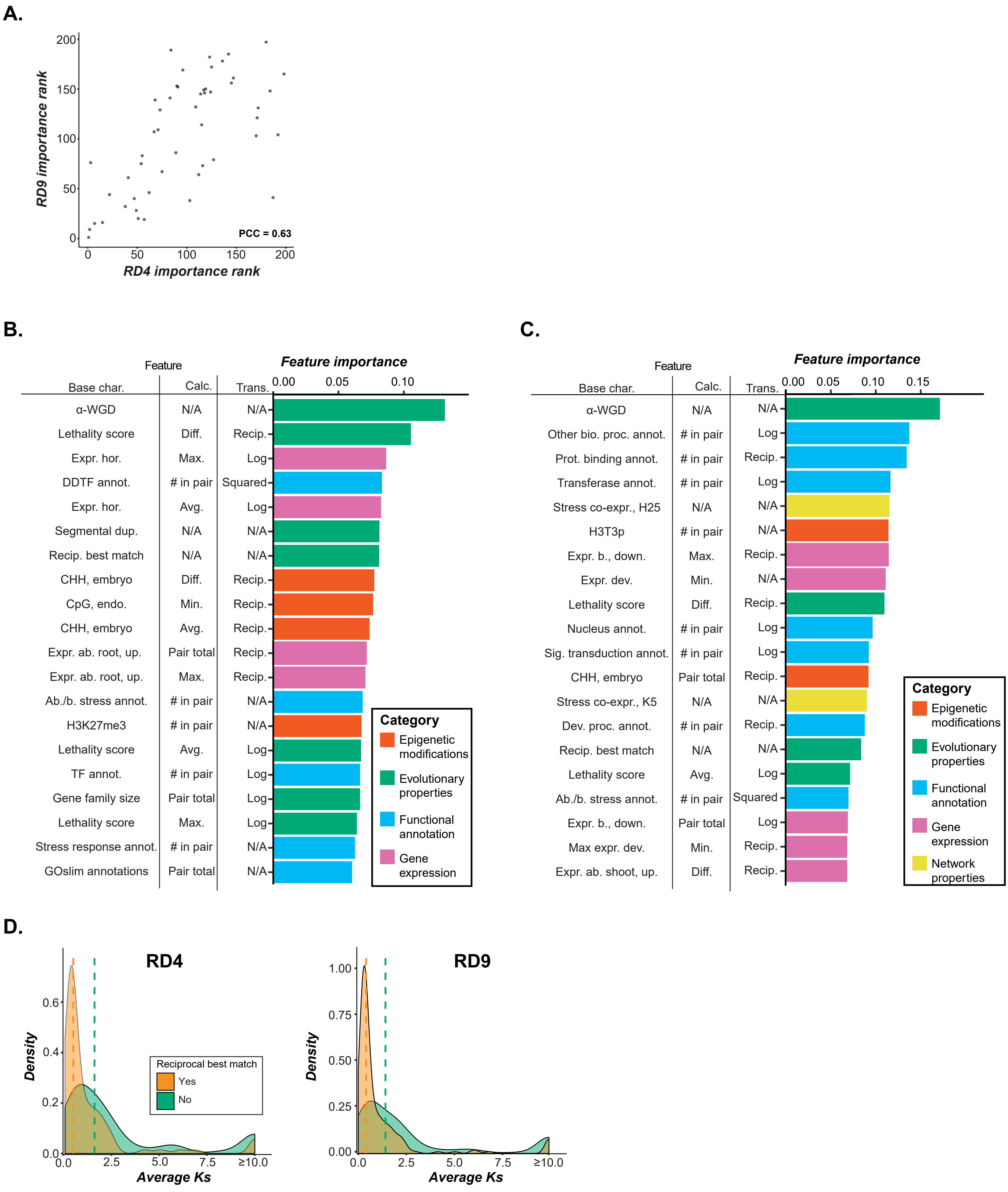
A.
RD9 importance rank
RD4 importance rank
PCC = 0.63
0
50
100
150
200
B.
Feature
Base char.
Calc.
Trans.
Feature importance
0.00
0.05
0.10
α-WGD | N/A | N/A
Lethality score | Diff. | Recip.
Expr. hor. | Max. | Log
DDTF annot. | # in pair | Squared
Expr. hor. | Avg. | Log
Segmental dup. | N/A | N/A
Recip. best match | N/A | N/A
CHH, embryo | Diff. | Recip.
CpG, endo. | Min. | Recip.
CHH, embryo | Avg. | Recip.
Expr. ab. root, up. | Pair total | Recip.
Expr. ab. root, up. | Max. | Recip.
Ab./b. stress annot. | # in pair | N/A
H3K27me3 | # in pair | N/A
Lethality score | Avg. | Log
TF annot. | # in pair | Log
Gene family size | Pair total | Log
Lethality score | Max. | Log
Stress response annot. | # in pair | N/A
GOslim annotations | Pair total | N/A
Category
Epigenetic modifications
Evolutionary properties
Functional annotation
Gene expression
C.
Feature
Base char.
Calc.
Trans.
Feature importance
0.00
0.05
0.10
0.15
α-WGD | N/A | N/A
Other bio. proc. annot. | # in pair | Log
Prot. binding annot. | # in pair | Recip.
Transferase annot. | # in pair | Log
Stress co-expr., H25 | N/A | N/A
H3T3p | # in pair | N/A
Expr. b., down. | Max. | Recip.
Expr. dev. | Min. | N/A
Lethality score | Diff. | Recip.
Nucleus annot. | # in pair | Log
Sig. transduction annot. | # in pair | Log
CHH, embryo | Pair total | Recip.
Stress co-expr., K5 | N/A | N/A
Dev. proc. annot. | # in pair | Recip.
Recip. best match | N/A | N/A
Lethality score | Avg. | Log
Ab./b. stress annot. | # in pair | Squared
Expr. b., down. | Pair total | Log
Max expr. dev. | Min. | Recip.
Expr. ab. shoot, up. | Diff. | Recip.
Category
Epigenetic modifications
Evolutionary properties
Functional annotation
Gene expression
Network properties
D.
RD4
Density
0.0
0.2
0.4
0.6
Reciprocal best match
Yes
No
0.0
2.5
5.0
7.5
≥10.0
Average Ks
RD9
Density
0.00
0.25
0.50
0.75
1.00
0.0
2.5
5.0
7.5
≥10.0
Average Ks

Figure S6

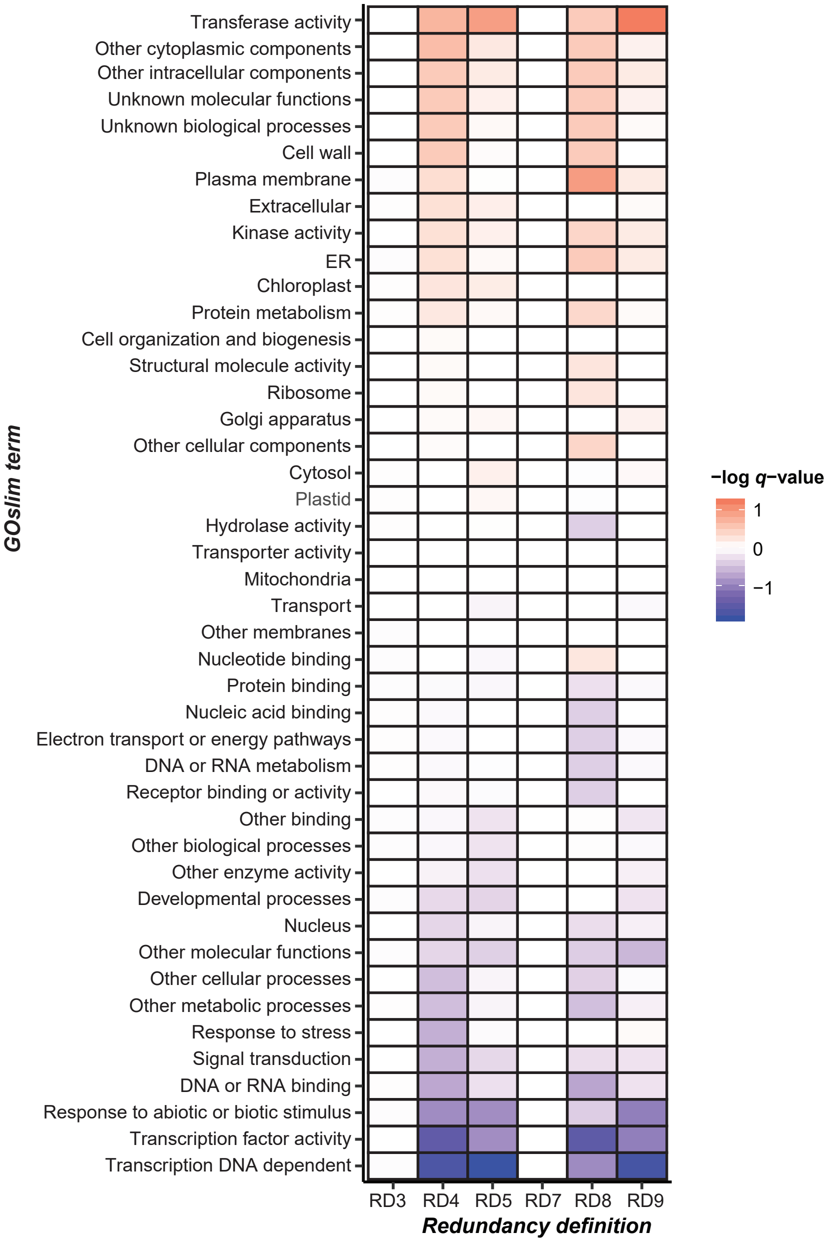
GOslim term
Transferase activity
Other cytoplasmic components
Other intracellular components
Unknown molecular functions
Unknown biological processes
Cell wall
Plasma membrane
Extracellular
Kinase activity
ER
Chloroplast
Protein metabolism
Cell organization and biogenesis
Structural molecule activity
Ribosome
Golgi apparatus
Other cellular components
Cytosol
Plastid
Hydrolase activity
Transporter activity
Mitochondria
Transport
Other membranes
Nucleotide binding
Protein binding
Nucleic acid binding
Electron transport or energy pathways
DNA or RNA metabolism
Receptor binding or activity
Other binding
Other biological processes
Other enzyme activity
Developmental processes
Nucleus
Other molecular functions
Other cellular processes
Other metabolic processes
Response to stress
Signal transduction
DNA or RNA binding
Response to abiotic or biotic stimulus
Transcription factor activity
Transcription DNA dependent
RD3
RD4
RD5
RD7
RD8
RD9
Redundancy definition
−log q−value
1
0
−1

Figure S7

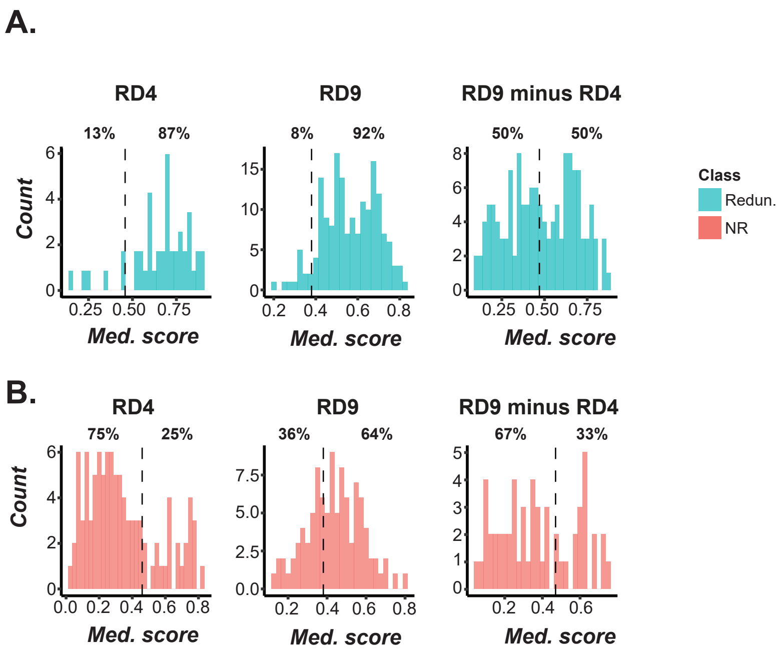
A.
RD4
RD9
RD9 minus RD4
13%
87%
8%
92%
50%
50%
Count
Med. score
Class
Redun.
NR
B.
RD4
RD9
RD9 minus RD4
75%
25%
36%
64%
67%
33%
Count
Med. score

Figure S8

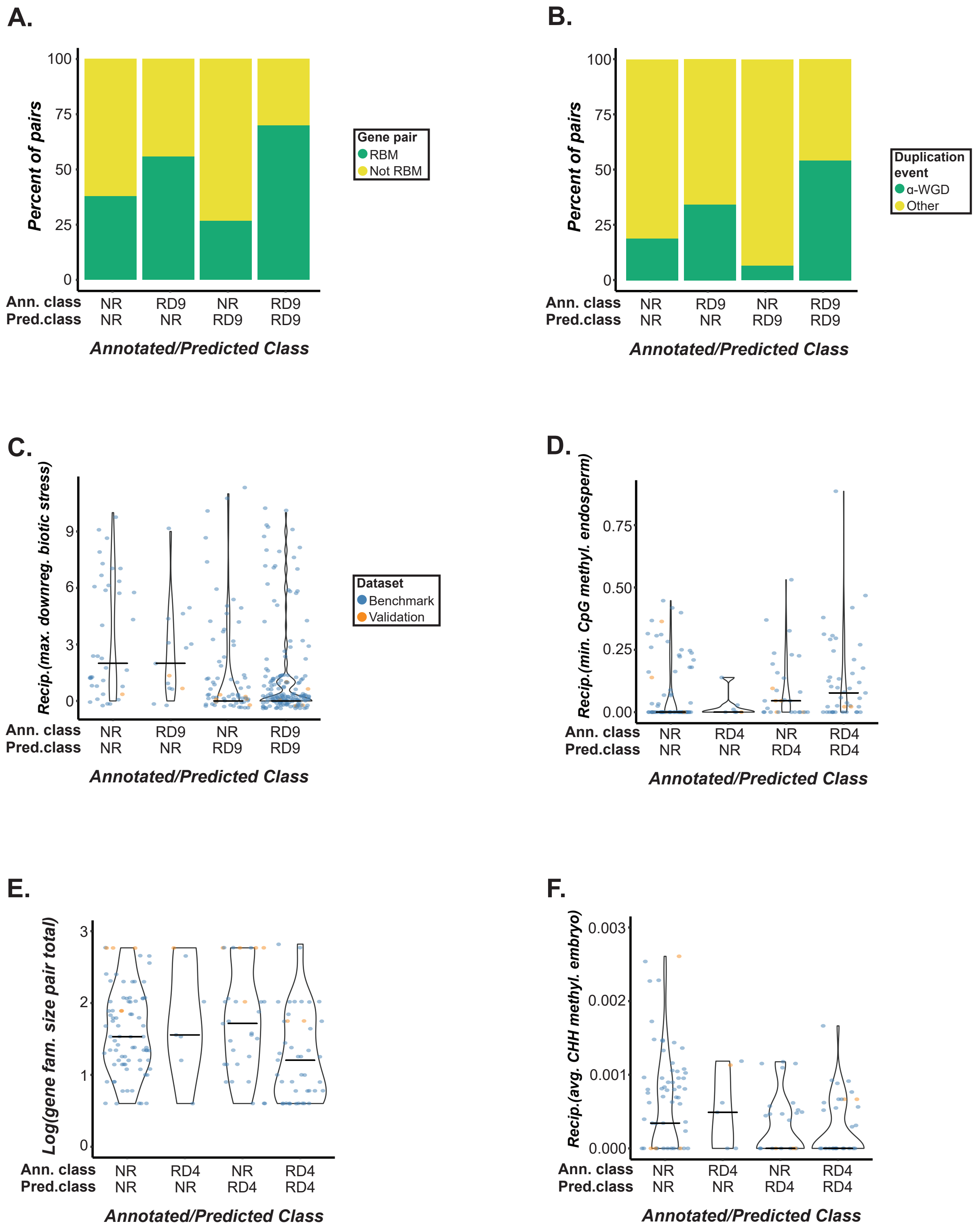
A.
100
75
50
25
0
Percent of pairs
Gene pair
RBM
Not RBM
Ann. class NR RD9 NR RD9
Pred.class NR NR RD9 RD9
Annotated/Predicted Class
B.
100
75
50
25
0
Percent of pairs
Duplication event
α-WGD
Other
Ann. class NR RD9 NR RD9
Pred.class NR NR RD9 RD9
Annotated/Predicted Class
C.
9
6
3
0
Recip.(max. downreg. biotic stress)
Dataset
Benchmark
Validation
Ann. class NR RD9 NR RD9
Pred.class NR NR RD9 RD9
Annotated/Predicted Class
D.
0.75
0.50
0.25
0.00
Recip.(min. CpG methyl. endosperm)
Ann. class NR RD4 NR RD4
Pred.class NR NR RD4 RD4
Annotated/Predicted Class
E.
3
2
1
0
Log(gene fam. size pair total)
Ann. class NR RD4 NR RD4
Pred.class NR NR RD4 RD4
Annotated/Predicted Class
F.
0.003
0.002
0.001
0.000
Recip.(avg. CHH methyl. embryo)
Ann. class NR RD4 NR RD4
Pred.class NR NR RD4 RD4
Annotated/Predicted Class
